# Emotional responses in Papua New Guinea show negligible evidence for a universal effect of major versus minor music

**DOI:** 10.1371/journal.pone.0269597

**Published:** 2022-06-29

**Authors:** Eline Adrianne Smit, Andrew J. Milne, Hannah S. Sarvasy, Roger T. Dean

**Affiliations:** 1 Department of Linguistics, University of Konstanz, Konstanz, Germany; 2 The MARCS Institute for Brain, Behaviour and Development, Western Sydney University, Milperra, NSW, Australia; 3 ARC Centre of Excellence for the Dynamics of Language, Australian National University, Canberra, ACT, Australia; Abertay University, UNITED KINGDOM

## Abstract

Music is a vital part of most cultures and has a strong impact on emotions [1–5]. In Western cultures, emotive valence is strongly influenced by major and minor melodies and harmony (chords and their progressions) [6–13]. Yet, how pitch and harmony affect our emotions, and to what extent these effects are culturally mediated or universal, is hotly debated [2, 5, 14–20]. Here, we report an experiment conducted in a remote cloud forest region of Papua New Guinea, across several communities with similar traditional music but differing levels of exposure to Western-influenced tonal music. One hundred and seventy participants were presented with pairs of major and minor cadences (chord progressions) and melodies, and chose which of them made them happier. The experiment was repeated by 60 non-musicians and 19 musicians in Sydney, Australia. Bayesian analyses show that, for cadences, there is strong evidence that greater happiness was reported for major than minor in every community except one: the community with minimal exposure to Western-like music. For melodies, there is strong evidence that greater happiness was reported for those with higher mean pitch (major melodies) than those with lower mean pitch (minor melodies) in only one of the three PNG communities and in both Sydney groups. The results show that the emotive valence of major and minor is strongly associated with exposure to Western-influenced music and culture, although we cannot exclude the possibility of universality.

## Introduction

In Western cultures, major harmony and melodies are typically perceived or felt to be happy, while their minor versions are typically perceived or felt to be sad [6–13]. Although several possible origins have been proposed [10], there is no consensus over exactly how harmony and pitch affect *emotive valence* (which we define in this study as happiness and sadness) and to what extent these effects are culturally mediated or universal [2, 5, 14–20]. We apply the term *universal* to a process by which a musical feature influences affect and is not a result of *familiarity* (statistical learning of that feature’s prevalence in the participant’s listening history) or of *association* (statistical learning of that musical feature’s spatio-temporal proximity to affect-laden events in the participant’s listening history). Experiments conducted with Western participants have shown that emotions induced by psychoacoustic features related to the pitch content of the musical signal—such as roughness, harmonicity, spectral entropy, and mean pitch—are relevant not only for familiar musical examples, but also for unfamiliar microtonal stimuli [21–23]. Although this suggests that harmony and melody may have the capacity to communicate universally, mixed results have been obtained from prior experimental investigations in remote communities without easy access to mass media: Mafa, Cameroon [24]; Mebenzélé Pygmies, Congo [18]; Tsimane’, Bolivia [19]; Khow and Kalash, Pakistan [25, 26].

The first of these cross-cultural studies used short piano pieces composed to express the emotions ‘happy’, ‘sad’, and ‘scared’. These emotional categories were recognized by Mafa participants above chance, and tempo and mode (major/minor) correlated with their responses. However, the pieces covaried across a number of musical features and the independent effect of mode (e.g., after controlling for tempo) was not included in the analysis. The second study used orchestral classical and film music and found no similarities between pleasantness ratings made by Canadian and Mebenzélé participants, and no effect of major/minor on the latter group. In the third study, which presented intervals and chords, US participants’ pleasantness ratings were predicted by traditional Western music-theoretical characterizations of consonance or dissonance; the Tsimane’ participants’ ratings were not. The fourth study examined dimensional ratings of valence, energy and dominance as well as of four basic emotions (joy, anger, sadness and fear) of short Western and non-Western musical harmonizations in different styles with participants from the UK and from two remote communities in Pakistan (the Khow and Kalash): mode significantly impacted valence ratings of the UK participants, but not of the Kho and Kalash participants (as explained by Athanasopoulos et al. [25], the tribe is referred to as Khow, whereas the people are named Kho). A connected study (from the same authors and the same population) testing positive and negative affect of isolated musical chords showed a cross-cultural aversion for dissonant clusters but a varied preference for major triads [26]. Although only the first of these studies hints at the presence of a universal effect of major/minor on perceived emotion, it is notable that none of them has taken a Bayesian approach. This is important, as a lack of evidence for a particular effect does not mean that there is no effect. It simply means that there may not be sufficient data collected to detect an effect. Hence, traditional frequentist methods are mostly only able to reject, or fail to reject, a null hypothesis, Bayesian analyses are required to quantify evidence for the absence of an effect [27].

The experiment reported here was conducted across a number of remote and self-sufficient communities in Papua New Guinea (PNG). None of the communities has regular access to mass media. According to our analyses and local knowledge, traditional music is similar across these communities (see the S1 File). In each trial, their task was to choose which of two chord progressions, or which of two melodies, represented the greater feeling of happiness. Hence, like the Mafa study [24] and the Khow/Kalash study, the question asked of our PNG participants is about a basic emotion (happiness).

Despite years of research on the association between major/minor and basic emotions such as happiness, there is still no consensus on its origin. Here, we aim to further explore possible mechanisms of this association in communities with varied exposure to Western music. Furthermore, by taking a Bayesian approach it is possible to assess evidence in favour of each effect as well as evidence for its practical absence [27].

Some of the chord progressions and melodies were in a major key, some in a minor key (the quantifications of major and minor are elaborated later). In every community, except the one with minimal exposure to Western-like music, we find strong evidence that major harmony induces greater happiness than minor, although less decisively than for Sydney participants. For melodies there is a very strongly evidenced effect of major and minor in one of the PNG communities, and in Sydney. The estimates are not sufficiently certain, however, to rule out a universal effect of major and minor in either cadences or melodies.

We can theorize at least two broad classes of culture-dependent mechanisms that potentially mediate the effect of musical features (such as major versus minor) on emotion [28, 29]. The first is *familiarity*. Stimuli that an individual has heard many times before—notably, over a lifetime of experience—are often preferred and can signify positive valence, perhaps because familiar events have greater perceptual fluency so take up fewer cognitive resources. Clearly, this is a culture-dependent mechanism because the musical sounds common in one culture may be uncommon in another (although due to cultural globalization, musical experiences are becoming rapidly homogenized hence the urgency for experiments such as reported here) [30].

The second is *associative conditioning*. Given consistent spatio-temporal pairings between musical features and valenced events, those musical features may become imbued with the associated valence [17]. For example, a person familiar with movies using Western music will more frequently hear major harmony in positively valenced scenes and minor harmony in negatively valenced scenes than the other way round.

An example of a musical feature that may influence emotion via a culture-independent mechanism is the mean pitch of the tones in a piece (which would differ between major and minor versions). Mean pitch has been found to influence emotional responses to music [10, 12, 22, 31–33], often showing a positive relationship with valence [9, 22, 33, 34]. This could be due to an innate bias—in both animal sounds and human vocalizations, high pitches are often associated with friendliness or submission, whereas low pitches are related to threat or aggression [35, 36]. Although pitch range of vocal expression in mammals is mostly associated with affective arousal rather than valence [37], subtle pitch height differences between musical stimuli have been associated with changes in perceived valence in human listeners. In two recent studies [22, 33], the association between pitch height and valence has been found for Westerners listening to chords from an unfamiliar microtonal (Bohlen-Pierce) [38] tuning system, which is suggestive that pitch height’s effect is independent of a listener’s musical culture [22]. However, these findings are not sufficient to establish whether this effect is truly universal. It may simply be that a culturally learned association between pitch height and valence is carried over from the familiar musical system to the unfamiliar system. Additionally, it must be noted that the pitch height differences between musical stimuli stimuli may be subtle compared to those in vocal expression.

Similar arguments hold for the emotional implications of major and minor harmony, which differ in psychoacoustic properties such as harmonicity, spectral entropy, and roughness. All else being equal, major chords have higher harmonicity and lower spectral entropy than minor chords, which should support the former’s perceptual fluency because, regardless of their familiarity, they are intrinsically simpler [39, 40]. Hence, these are possible routes for a culture-independent effect of major/minor on valence. Recent studies have demonstrated that each of these three psychoacoustic features predict the perceived valence of chords in an unfamiliar tuning system [22, 23]; but, as before, this may just be a carry-over from culturally learned associations between major and minor chords and valence.

In this study, we focus on how self-reported valence of harmony is affected by cadence type (major versus minor) and *mean pitch*, and how self-reported valence of melodies is affected by *mean pitch*. To ascertain whether any of these features are mediated through universal mechanisms, we conducted an identical experiment in five remote PNG communities (with participants from seven different villages) with limited but differential experience of Western music, and in two Sydney cohorts with considerable, but still differing, levels of experience with Western or Western-like music. (Our definitions for Western and Western-like music, major and minor, and other musicological terms are provided in the S1 File.)

The first participant group completing the experiment comprised inhabitants of Towet village, in the Uruwa River Valley, Saruwaged Mountains, Morobe Province, PNG, and were tested during a three-week field trip to the area. After the research team left, local research assistants hiked to the villages of Mup, Mitmit, Kotet, and Yawan, and repeated the experiment on participants from those villages as well as from Bembe and Worin.

The Uruwa River valley is a cloud forest area accessible only by small plane or, for locals, an arduous three-day hike. Between the valley floor and the surrounding mountains, elevation ranges from sea level to peaks of 4,000 m. Villagers are expert farmers and lead self-sufficient lives, without mains electricity or an internal market economy. Mobile phone coverage reached the region in mid-2015, but internet access over the mobile phone network is nearly impossible. A dialect of the Nungon language is spoken in all the villages [41]. Additional information on the area—geography, maps, musical traditions, linguistic, and historical—is provided in the S1 File.

As summarized below, there are three genres of music present in different, sometimes overlapping, parts of the Uruwa valley: traditional non-Western-style song; Western-influenced *stringben* (which means ‘string band’ in Tok Pisin, the PNG lingua franca); and Western church hymns.

Across much of the region, traditional songs are performed at gatherings or specific occasions, such as a successful hunt or the blessing of crops, and are commonly accompanied with a *uwing* drum, which is a wooden hourglass-shaped drum with a single animal-skin head [42], or handmade flutes. Detailed analysis of six recordings of songs in a non-Western style is provided in the S1 File; in summary, the melodies are monophonic (sung solo or in unison) and rarely exceed a perfect fifth in range. There is typically a ‘focal’ pitch, which is sung more than any other. With the possible exception of a small whole tone (about 1.75 semitones), interval sizes are inconsistent between the songs, and are somewhat inconsistent within each performance. They do not typically conform with Western intervals. The aggregated pitch content of each song (e.g., overall pitch range, number of pitches, or mean pitch relative to focal pitch) does not correlate to the emotive content of lyrics or performative contexts, or the village of origin.

Due to missionary involvement, villages in the Uruwa area have adopted either the Lutheran or the Seventh-day Adventist (SDA) church, although the extent of involvement with either varies both within and between the villages. Bembe, Mitmit, Mup, and Worin residents are mostly Lutherans who attend church regularly and hear and perform hymns on Sundays. Based on local people’s reports, and literature describing the Lutheran movement in urban areas of Morobe Province, Lutheran church service hymns are a mixture of traditional and *stringben* sung melodies, often accompanied by guitar or ukulele in the *stringben* style. *Stringben* songs are mostly in a major key with generally major chords [43, 44]. Indeed, our analysis of Lutheran hymns recorded in Towet, which shows they comprise 92% major chords and 8% minor (detailed in the S1 File). Residents of these villages also probably engage in *stringben* and traditional music during the week.

People from Towet are mostly church-going SDA adherents who hear and perform hymns from the *SDA Hymnal* [45] at services on Friday nights and Saturdays, and in occasional morning and evening services during the week. The hymns are mostly sung in English, and the analysis in the S1 File shows that a substantial majority of their keys and chords are major (86% major chords, 12.5% minor, 1% diminished, 0.5% other), and there is no evident association between the valence of the words and the harmony (major/minor). There is very limited partaking in *stringben* or traditional music during the week (it is frowned on). Kotet and Yawan villages lack their own Lutheran churches (Kotet’s was demolished in 2011) and are too far from Worin or Mup to make regular church attendance practicable, but do have SDA churches. This means that *stringben* and traditional music are likely played or heard sporadically only, while SDA followers hear and sing SDA hymns weekly.

The above implies three groups of Uruwa participants with different levels or types of exposure to Western or Western-like music. The *minimal exposure group* comprises non-church-goers, and Lutherans in Kotet/Yawan—they have had only sporadic experience of Western-like music for at least seven years prior to the experiment. The *Lutheran exposure group* comprises all other Lutheran church-goers—they have regular exposure to major harmonies and melodies but less exposure to minor. The *SDA exposure group* comprises all SDA church-goers—they have regular exposure to major harmonies and melodies and, compared with the Lutherans, a slightly wider palette of Western harmonies; they have less regular exposure to traditional music than the other two groups. Importantly, none of the Uruwa participants have had regular exposure to conventional Western associations between musical features and emotion.

In contrast, the Sydney participants are all well-steeped in such associations, and have considerable exposure to Western music. Of the two Sydney groups (non-musicians and musicians, the latter having at least five years training or performance experience), the musicians generally have greater exposure to, and knowledge of, Western music and its cultural associations, and have more refined audition skills (for example pitch discrimination). Therefore, along with the three Uruwa groups, we have a total of five participant groups with differing levels and types of exposure to Western or Western-like music and its cultural embeddings. These five groups serve as the basis of our statistical analysis and its interpretation.

To briefly summarise the stimuli and procedure, every participant was presented with 12 different pairs of major and minor *cadences* (chord progressions traditionally used in Western music to unambiguously assert a major or minor key) and 30 different pairs of melodies in different modes (Phrygian, Æolian, Dorian, Mixolydian, Ionian, and Lydian). The cadences had a variety of tonic pitches, hence any effects of major versus minor and mean pitch can be assessed independently. The melodies all had the same ‘tonic’ (sounded by a drone), hence changes in their modes cannot be disambiguated from changes in their mean pitch.

For the Sydney participants, we expected major cadences to be more likely identified as happy when compared with minor cadences, and for cadences with higher mean pitch to be more likely chosen as the happy one (even after controlling for cadence types; for melodies, we expected results similar to those of Temperley and Tan [9]—the melodic subject with the higher mean pitch is more likely identified as the happy one, but unfamiliar modes (notably Lydian and Phrygian) are also less likely identified as the happy one. For the Uruwa River Valley participants, we anticipated the similarity of their responses to the Sydney participants would be positively associated with their familiarity with Western music. Based on these predictions, we tested the following hypotheses on self-reported happiness for each group of participants:

1. A positive effect of major versus minor for cadences only, adjusting for mean pitch.
2. A positive effect of an increase in mean pitch for cadences only, adjusting for major versus minor.
3. A positive effect of an increase in mean pitch for melodies only.
4. A positive effect of an increase in mean pitch on cadences and melodies combined.

## Methods

### Participants (Uruwa River Valley, Papua New Guinea)

The first participant group consisted of adults (above 18 years old) tested in the Uruwa River Valley, Saruwaged Mountains, Morobe Province, Papua New Guinea. A total of 170 Uruwa participants took part in the experiment and 24,445 observations were collected (*n* = 170, female = 85, mean age = 33.3 years, s.d. = 13.9 years (one participant who did not provide interview data was removed from the model, leading to a total of 169 participants). Participants were inhabitants of the following villages: Towet, Worin, Bembe, Mitmit, Mup, Kotet, and Yawan. Although some are relatively close to each other, the difficult mountainous terrain means that walking between them takes locals between 30 minutes and many hours (and substantially longer for those unaccustomed to the environment, such as the research team). Due to time constraints and the significant walks required to reach some villages, the experiments in Mup, Mitmit, Kotet, and Yawan were conducted by local research assistants Namush Urung, Ben Waum and Nathalyne Ögate.

Test dates, location (including photographs of the set-up), and participant demographics are detailed in the S1 File. The sample size was chosen to be as large as possible depending on the availability of adults in the villages. Participants were rewarded with 50 PGK (roughly 20 AUD). The study is in accordance with relevant guidelines and regulations. Written informed consent was obtained from all participants prior to the start of the experiment. The three local research assistants assisted in the researchers’ presence and independently conducted these experiments in the researchers’ absence, translated from English to Nungon and verbally explained and translated the participant information sheet, consent form, and the experimental procedure upon which participants could provide their written consent. Participants, the research assistants, as well as other community members who were involved in helping to organize the research trip, were financially compensated for their work in local currency at a rate suitable for the local context at an hourly/daily rate equivalent to those paid by the third author for other research activities (see [46, 47]. We provide more details regarding the ethical considerations of the research in the S1 File.

Data were removed blockwise by trial type (cadence, melody, intervals, or triads) when a participant always answered ‘one’ or ‘two’ or followed an alternating pattern of ‘one’ and ‘two’ because such a response pattern suggests that task instructions were not followed or that participants applied a specific strategy to answer that was unrelated to their actual self-reported happiness (see the S1 File). Having said that, results changed only slightly compared with models fitted to all the data, and no hypotheses came close to transitioning between strongly and not-strongly evidenced.

### Participants (Sydney, Australia)

The second participant group consisted of musicians (*n* = 19, female = 11, mean age = 27.3 years, s.d. = 8.7 years) and non-musicians (*n* = 60, female = 50, mean age = 21.8 years, s.d. = 6.4 years) tested at Western Sydney University in Australia. The non-musicians were undergraduate psychology students recruited through the Western Sydney University’s online research participation system (SONA), whereas the musicians were recruited by adverts and word-of-mouth from the Sydney area. Musicians were defined as having received more than 5 years of formal training in music or music theory. All participants were above 18 years old. After data exclusions resulting from patterned responses or no responses (as detailed in the S1 File), no participants were removed but one block of cadence trials was removed. Non-musician participants were rewarded with course credit and musicians with 20 AUD, as they were separately recruited. Written informed consent was obtained from all participants prior to the start of the experiment.

### Materials

The first stimulus was preceded by a recording of the Nungon word for ‘one’, the second by ‘two’. Uruwa participants then responded to the question ‘when you hear which tune, are you happy?’ to which the answer is ‘one’ or ‘two’ (this phrasing sounds awkward in English translation, but it follows the standard sentence structure used in Nungon). Hence, this question requires the comparison of levels of a basic emotion. A forced-choice design, rather than a Likert or continuous scale, was appropriate because the Nungon verb used in the question already entails ‘be very happy’, relative to the equivalent in English, so attempting to modify it with intensifiers would not make much sense. Furthermore, members of these communities are not familiar with scalar or ordinal rating tasks.

Each cadence trial consisted of two successive cadences consisting of two successive chords, each cadence in a distinct key, from the set B major, C major, D♭ major, B minor, C minor, and C♯ minor. They were presented in these different keys to disambiguate any effect of mean pitch from any effect of cadence type (major versus minor). Out of all possible ordered pairs from the above set, each participant heard one of two subsets of 12, each of which ensured every key was heard the same number of times. Three melodic *subjects* (melodies specified by scale degree rather than pitch) were presented in 6 different modes of the diatonic scale (Phrygian, Æolian, Dorian, Mixolydian, Ionian, Lydian, listed from lowest to highest mean pitch or, equivalently, from most minor to most major) to test whether mean pitch impacts valence in a melodic context. Every mode had the same tonic, which was asserted with a low C octave sounding simultaneously with the melodies, and every melody ending on that pitch class. Each trial was a sequence of two versions of the same melodic subject, each in one of those modes. All 30 ordered pairs of distinct modes were used. A more detailed description of the stimuli is available the S1 File; there were also other stimuli related to perceptions of harmonic stability, which are not analysed here.

Between participants, and between the cadence and melody blocks, two sample sets were used: a vocal choir or a string quartet. For the Uruwa participants, bowed strings are not physically found in this region of PNG and thus are an unfamiliar timbre. Although not the primary purpose of this study, differing responses to these two timbres might, amongst other things, indicate that timbral familiarity moderates responses.

Stimuli were generated in Max 7 (Cycling ’74) and presented in PsychoPy v.3.0.2. Stimuli consisted of pairs of intervals, triads, cadences, and melodies and were created in a vocal and an instrumental timbre (only the cadences and melodies are reported here). The vocal timbre was sampled from the voice sample library ‘Voices of Rapture’ (Soundiron) with the soprano (S) singing ‘Oh’ and alto (A), tenor (T) and bass (B) singing ‘Ah’. The instrumental timbre was sampled from ‘Solo Strings Advanced’ (Dan Deans) with the S as violin 1, A violin 2, T viola and B cello, which is a standard string quartet. Cadences used SATB (soprano, alto, tenor, bass); melodies A for the melody and T plus B for the octave C drone; intervals were T plus B; triads were A, T and B. The MIDI was generated in Max 7, sent to the above sample sets played in the software Kontakt 5 (Native Instruments), with each of the four instruments panned to a unique left-right position much as would be found in a commercial string quartet recording, and saved as wav files. Stimuli were created in 12 different versions, counterbalancing between the two timbres per stimulus category and with random pitch transpositions for the intervals and triads, enabling us to test as many pairs of intervals, triads, cadences and melodies as possible. These 12 versions were varied between participants. The 144 stimuli were presented over five blocks. Examples of the stimuli can be downloaded from https://osf.io/c3e9y/files/.

The order of the blocks was as follows: intervals (30 trials), cadences (12 trials), intervals (30 trials), melodies (30 trials), triads (42 trials). Block order was fixed, but stimulus presentation within blocks was randomized. Participants were asked to decide which stimulus represented the feeling of happiness (‘one’ or ‘two’) for cadences and melodies, and which stimulus was ‘finished’ (‘one’ or ‘two’) for intervals and triads. The questions for this study were designed to accommodate the cultural and linguistic differences between Western listeners and PNG listeners.

In consultation with community leaders and the research assistants in Towet, language experts at the Summer Institute of Linguistics in Ukarumpa, PNG and based on H.S.S.’s years of research on the Nungon language, we decided to use paired stimuli with binary forced choice between the first or second stimulus using the Nungon word *dongko-ha-rok* (‘be.happy-present.tense-you’; ‘you are happy’) for the happiness question (as detailed in the main text) and *buret-ta-k* (‘finish-present.tense-s/he/it’; ‘it has finished’) for the finished question. (The data from the ‘finished question’ will be reported elsewhere.) A third rating option for ‘neither stimulus’ was not included because it adds to the complexity of the task, and because a neutral response option may encourage a lack of engagement with the task.

### Procedure (Uruwa River Valley, Papua New Guinea)

As with many cross-cultural adaptations of WEIRD (Western, Educated, Industrialized, Rich, and Democratic) research paradigms [48, 49], there are multiple components to comprehension of the task for our participants. We identified: (a) comprehension of the question being asked (‘When you hear which tune, are you happy?’); (b) familiarity with the notion that a melody could make them ‘feel happy’; and (c) understanding of the expected process a participant should go through to answer the question each time (evaluate the two musical fragments and choose the one that makes them feel happy).

(a) We have no doubt that participants understood the question at a simple linguistic level. H.S.S. is fluent in Nungon and wrote the authoritative 627-page reference grammar of the language [41]. The question was kept short and maximally simple to ensure that there was no ambiguity in it. H.S.S. discussed the wording of the question at length (in Nungon) with J.J., S.G., and L.Ö., who helped organize our stay in Towet and further with research assistants B.W., N.U., and N.Ö., to ensure that it was maximally felicitous. For ‘happy’, we considered framing it as ‘is the person making the music happy?’, but decided this offered too much space for misunderstanding.
  In sum, the question was succinct and well-formed in Nungon, structured as a typical short Nungon sentence. H.S.S. indicated that the wording left no room for ambiguity, and this was affirmed by the six consulting community members.
  After such lengthy consultation with multiple community members, in Nungon, about the wording of the question, we did not expect participants to misunderstand it. This was borne out by the experimentation, during which we saw no indication that participants did not understand the question. They responded appropriately and promptly, ‘one’ or ‘two’; we never observed anyone asking for the question to be repeated so that they could understand how to respond to it, or responding with something other than ‘one’ or ‘two’.
(b) In H.S.S’s experience, and in that of senior PNG ethnomusicologist Don Niles (p.c., 2019), the Uruwa communities and similar communities elsewhere in PNG do not habitually discuss novel wordless melodies or cadences as evoking emotion alone. It is much more common in their experiences for the words of songs to be described as evoking emotions. Songs can also be linked to celebratory or somber occasions. We thus expected that participants might be puzzled by the demand in the task here that they feel happiness on hearing a short, contextless melody without words. Thus, the question itself could be understood, but a participant might still not be able to comprehend how they were supposed to choose their response (‘one’ or ‘two’).
  We presented our plans for this research project in a Synergy seminar at the Summer Institute of Linguistics—PNG branch (in Ukuraumpa, Eastern highlands Province, PNG), and received helpful feedback from longtime fieldworkers in PNG, including Andrew Grosh and Matthew Taylor, on the best ways to frame the question. What could puzzle participants here would be not how a melodic fragment could make them feel happy but how they would be able to decide without knowing what song it comes from.
(c) Presumably, participants could understand the literal meaning of the question, and even be familiar with the notion being asked about, but still not understand how they were expected to respond to relatively rapid-fire questions and stimuli presentations in a psychology experiment. It is widely known that there are cultural differences in the ways questions are used in discourse and in the expected ways of responding to them. Some Aboriginal communities in Australia, for instance, are known to have a cultural dispreference for asking and being asked direct questions, and to sometimes display so-called ‘gratuitous concurrence’ (replying ‘yes’ to positive questions and ‘no’ to negative questions, regardless of veracity [50]) when interacting with non-Aboriginals. Our question did not use yes/no responses to positive/negative questions.
  For the Uruwa community, participants lack familiarity with the notion of being asked the same question repeatedly about a series of musical fragments, with the accompanying expectation that they quickly evaluate each on its own merits. This lack of familiarity might not translate into difficulty; some participants might understand and enthusiastically take up the novel task. But the lack of familiarity could mean that other participants might respond at random, or use patterned responses, without understanding that they were not ‘supposed to’ do this in this type of experimentation.

For the first set of participants in PNG, with the experimenters present, the experiment was conducted on the ground floor of a two-storey wooden building in Towet. For the experiments conducted in other villages, the local research assistants were instructed to test in a quiet location. Participants were seated in front of one of the researchers or research assistant and listened to the stimuli through headphones (Audio-Technica M50x or KOSS UR20). As outlined in the main text, every trial followed the same procedure. First, participants heard the word *ingguk* (‘one’), followed by stimulus 1, then the word *yoi* (‘two’), followed by stimulus 2. After stimulus presentation, participants were asked verbally, in Nungon: ‘When you hear which tune, are you happy?’. Answers were recorded in PsychoPy by the experimenters or the local research assistants. Prior to the experiment, participants were presented with four practice trials with both questions to ensure complete understanding of the task. An interview in Nungon was conducted after the experiment to obtain more information about the participants’ cultural and musical background (see the S1 File for the specific interview questions). Questions were asked orally in Nungon by local research assistants and responses were recorded by them or the researchers. The interviewers would interpret the responses as matching a particular choice of those available.

The principal purpose of the questionnaire was to assess musical exposure. However, many of the factors, which were coded from the responses, and all of their interactions, had levels with an insufficient number of participants to make generalization safe. Furthermore, it was apparent that at least some of the responses were inaccurate; for example, participants who were known by other community members to not regularly attend church said they did. Hence, we only included village, church denomination and church attendance in the models, as these were the most reliable answers to define exposure. Estimates are thus at the individual level, based on participant’s responses to the questions of church attendance, village and church type, which led to the three groups discussed in the main text.

### Procedure (Sydney, Australia)

Participants in Sydney were tested in a soundproof room, seated behind a laptop and listened through headphones (Sennheiser HD 280 Pro, Beyerdynamic DT 770 Pro, or Sennheiser HD 650). The experimental task was explained by a research assistant, but participants indicated their answers themselves by pressing buttons on a keyboard. After the experiment, participants were asked to complete the Goldsmith’s Musical Sophistication Index questionnaire, which is designed to elicit information from participants regarding their engagement with music, self-reported listening, singing skills, musical training, and their emotional engagement with music [51]. Some additional questions related to their demographics and musical heritage were asked as well. Even though all participants lived in the metropolitan area of Sydney, many had a multicultural background. A breakdown of participants’ countries of origin (they were all residing in Australia) is provided in the S1 File). The vast majority of participants indicated they listen mostly to Western music genres, such as pop, classical, jazz, R&B, EDM, hard-style, rock, metal, indie, house and drum & bass. Only five participants indicated listening to non-Western music genres, which were Arabic music, classic Chinese music, classical music in other languages (Urdu, Pashto, Punjabi and Kashmiri), and Bollywood music. Of those five participants, only one (the Arabic music listener) did not indicate listening to any Western music genre.

Experiments were conducted in June/July 2019 (Towet village) and July/August/October 2019 (other villages, Western Sydney University). The study was approved by the Towet community leaders, the Papua New Guinea National Research Institute, and the Western Sydney University Human Research Ethics Committee (H13179).

### Statistical modelling and data analysis

#### Mean pitch

Mean pitch is calculated as the mean MIDI pitch of all pitches in the stimulus under consideration. For the melodies and cadences, the mean over all pitches in each stimulus in the pair is calculated. The difference in mean pitch—the predictor used in the models—is simply the mean pitch of the second stimulus minus the mean pitch of the first stimulus, and has semitone units.

#### Cadence type

Here, we refer to the type of cadence, which is either major or minor (see the S1 File for our definition of major and minor). In music theory, this could also be referred to as the cadence’s ‘mode’ but, to avoid confusion with the melody modes, we use ‘type’ for the cadences.

#### Mode

For melodies, ‘mode’ refers to one of the six different scales each melody was played in; here listed in ascending order of average pitch: Phrygian, Æolian, Dorian, Mixolydian, Ionian, and Lydian (Ionian is the common major scale, Æolian is the natural minor scale). To reduce the experiment’s duration, the rarely used Locrian mode was omitted (as in Temperley and Tan [9]).

#### Timbre

Timbre generally refers to the perceived quality or ‘colour’ of a sound and has been suggested to have an independent and robust effect on listener’s perception of emotion in music [2, 16, 52, 53], which is not driven by musical expertise [54]. For this experiment, we used two timbres (a vocal and a bowed string timbre). Timbre is used as a moderator of the main predictors in this experiment: *cadence type* and *mean pitch difference*.

**Fig 1:**
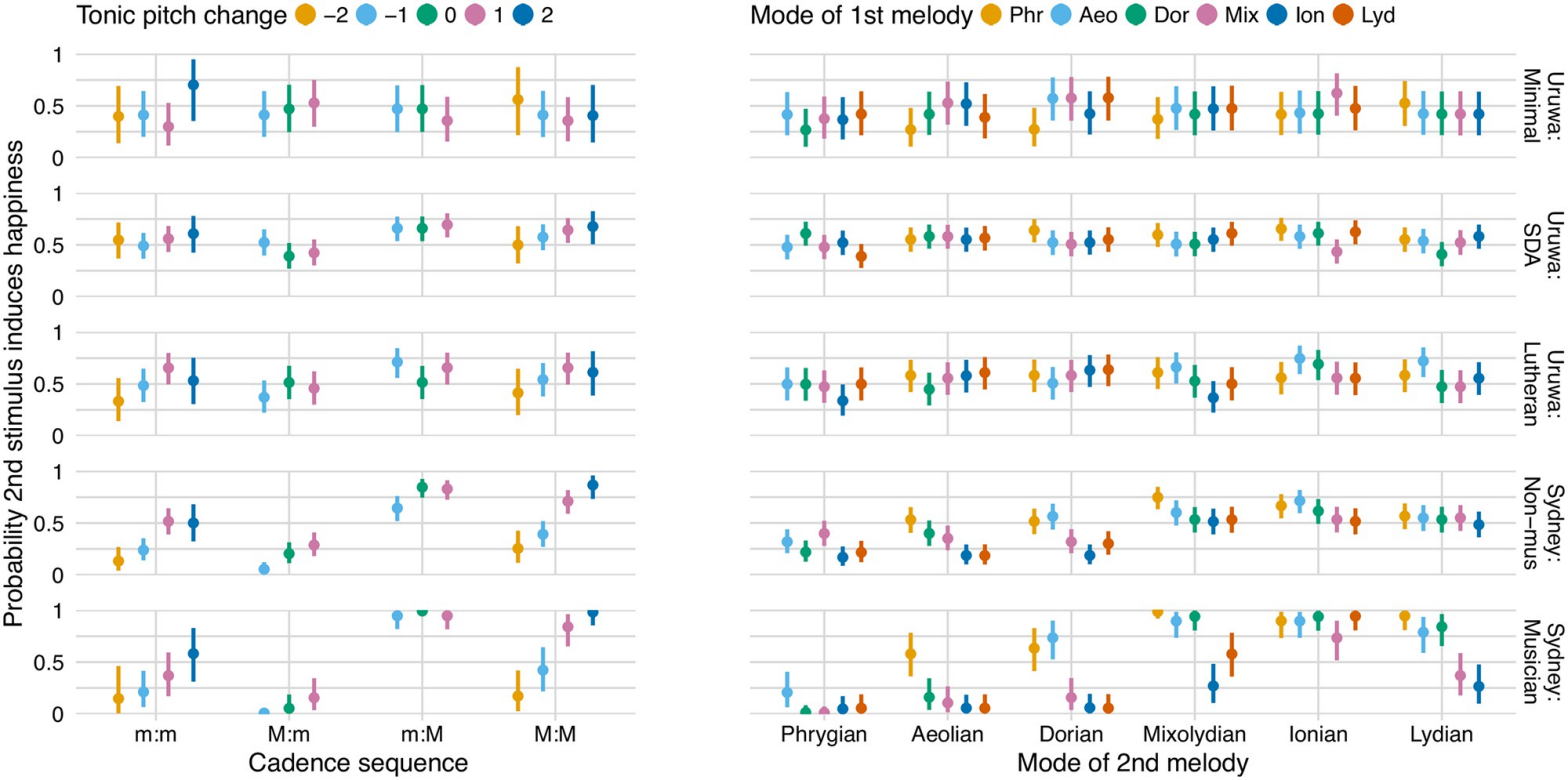
Visualizations of the effects of standard musicological categorizations across the five groups of participants. For each pair of cadences, the effects produced by their cadence sequence and their tonics’ semitone pitch difference; e.g., a C minor cadence followed by a B♭ major cadence has the cadence sequence ‘m:M’ and a tonic pitch change of −2 semitones. For each pair of melodies, the effect of their mode sequence (the Phrygian mode has the lowest mean pitch followed by Æolian, followed by Dorian, and so forth, up to Lydian, which has the highest mean pitch). The bars show the descriptive models’ 95% credibility intervals.

**Fig 2:**
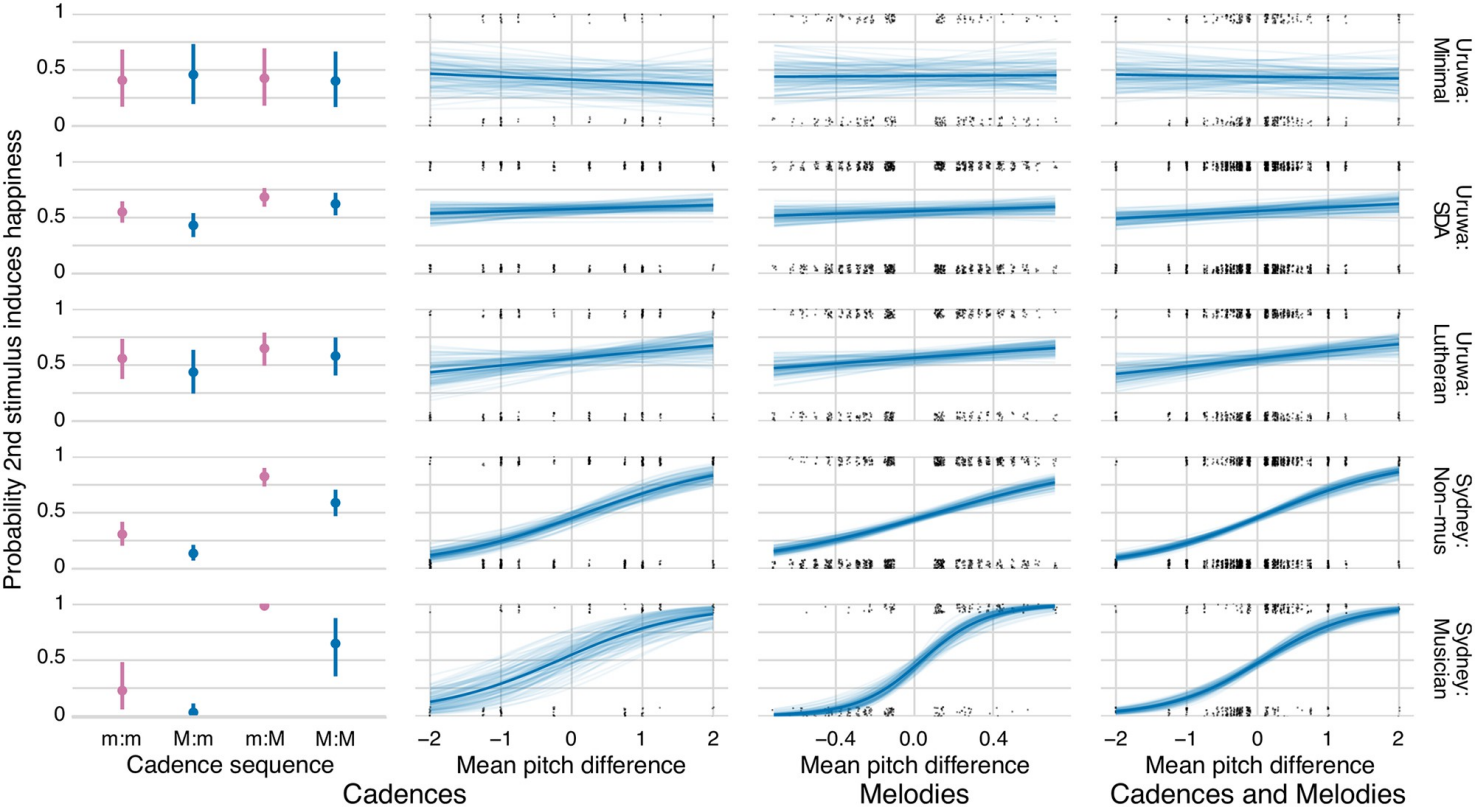
Visualizations of the models’ principal effects across the five groups of participants. For cadences: (a) the effect of their cadence sequence, adjusted for their mean pitch difference and timbre (the difference between the inner two posterior distributions corresponds to H1); (b) the effect of their mean pitch difference (semitones), adjusted for their cadence sequence and timbre (H2). For melodies: (c) the effect of their mean pitch difference (semitones), adjusted for timbre and melodic subject (H3). For cadences and melodies: (d) the effect of their mean pitch difference (semitones), adjusted for timbre (H4). For each cadence sequence plot, the posterior mean and 95% credibility intervals are shown. For each mean pitch difference plot, the posterior mean slope is shown with the thick line, while its uncertainty is visualized with 100 samples from the posterior distribution. The grey dots, which have been spatially jittered, show the observed values.

*Models and coding*. The data were analysed in R [55] using the brms package [56, 57], which is a front end for the Bayesian inference and Markov chain Monte Carlo (MCMC) sampler Stan [58]. Two types of Bayesian logistic models were run: descriptive models designed to summarize overall patterns in the data (visualized in Fig 1), and hypothesis-driven models designed to assess evidence both for and against the principal hypotheses (H1–H4) related to cadence type (major/minor) and mean pitch difference (see the Introduction), as well as their expected effect sizes (reported in Table 1, Fig 2, and the main text).

**Table 1:** Hypothesis tests and summaries of the models’ principal effects across the five groups of participants (the hypotheses are detailed in the main text). ‘Est’, ‘Q5%’, and ‘Q95%’ show the mean and 90% equal-tailed credibility interval for the effect of Min to Maj compared to Maj to Min (H1) or a one-semitone increase in mean pitch (H2–H4). This effect is expressed on the logit (log-odds) scale. ‘Evid.ratio’ is the odds that the effect is in the direction specified by the hypothesis, and ‘Post.p’ is the associated posterior probability of the hypothesis (*Evid.ratio* = *Post.p*/(1 − *Post.p*)). We consider evidence ratios > 10 to be strong evidence; > 30 to be very strong. ‘ROPE’ shows the probability that the effect is small enough to be practically equivalent to zero [27], which we define as being in the interval [−0.18, 0.18].

Exposure group by hyp.	Est.	Q5%	Q95%	Evid.ratio	Post.p	ROPE
*H1—Cadence type*						
Uruwa: Minimal	−0.14	−1.19	0.91	0.69	0.41	0.23
Uruwa: SDA	1.06	0.59	1.54	6665.67	1.00	0.00
Uruwa: Lutheran	0.90	−0.01	1.84	18.14	0.95	0.07
Sydney: Non-musician	3.47	2.71	4.31	>19999.00	1.00	0.00
Sydney: Musician	8.96	6.08	12.64	>19999.00	1.00	0.00
*H2—Cadences mean pitch*						
Uruwa: Minimal	−0.08	−0.40	0.23	0.49	0.33	0.62
Uruwa: SDA	0.08	−0.06	0.21	4.63	0.82	0.90
Uruwa: Lutheran	0.27	0.00	0.53	19.70	0.95	0.29
Sydney: Non-musician	0.91	0.67	1.17	>19999.00	1.00	0.00
Sydney: Musician	1.15	0.70	1.66	3999.00	1.00	0.00
*H3—Melodies mean pitch*						
Uruwa: Minimal	0.09	−0.52	0.69	1.46	0.59	0.37
Uruwa: SDA	0.22	−0.08	0.50	8.12	0.89	0.41
Uruwa: Lutheran	0.51	0.13	0.89	65.67	0.98	0.08
Sydney: Non-musician	2.06	1.59	2.52	>19999.00	1.00	0.00
Sydney: Musician	6.31	4.81	7.86	>19999.00	1.00	0.00
*H4—Cadences and melodies mean pitch*						
Uruwa: Minimal	−0.05	−0.28	0.18	0.56	0.36	0.78
Uruwa: SDA	0.14	0.03	0.25	54.25	0.98	0.74
Uruwa: Lutheran	0.27	0.09	0.45	120.95	0.99	0.20
Sydney: Non-musician	1.02	0.83	1.22	>19999.00	1.00	0.00
Sydney: Musician	1.56	1.27	1.88	>19999.00	1.00	0.00

For all models, every factor was sum-coded (also called deviation-coded), so the reported effect of any predictor is a *main effect*; that is, the mean effect of that predictor over all other factor levels, such as timbre or melodic subject. In the hypothesis-driven models, the mean pitch differences between the two stimuli were in semitone units ranging from −2 to + 2 for cadences; and from −0.71 to + 0.71 for melodies, with respective standard deviations of 1.17 and 0.34. The reported effect of a unit increase in any predictor represents an additive change on the logit scale, which is the logistic-distributed latent scale assumed—in any logistic model—to underlie participants’ binary decisions.

*Descriptive models*. In summary, the descriptive models’ predictors are basic musicological descriptions of the stimuli: the relationships between the keys and modes of the two stimuli in each trial. In order to present the data in as ‘raw’ a form as possible (in a Bayesian context), no random effects were included and the priors were approximately uniform on the probability scale. The data were categorized using standard musicological descriptions with a comprehensible number of levels: cadences were categorized by their sequences (minor to minor, minor to major, major to minor, and major to major) and the change in the tonic pitch from the first to second cadence; melodies were categorized by their sequence of modes.

In both models, all other features such as timbre or melodic subject (both of which were balanced by design) were not included and hence averaged over. More formally, in the descriptive models, we have the following predictors:

- *cad_mode1* ∈ {Min, Maj} (type of the first cadence)
- *cad_mode2* ∈ {Min, Maj} (type of the second cadence)
- *tonic_pitch_diff_cat* ∈ {‘−2’, ‘−1’, ‘0’, ‘1’, ‘2’} (semitone pitch difference of the two cadences’ tonics, coded as categories)
- *mel_mode1* ∈ {Phrygian, Aeolian, Dorian, Mixolydian, Ionian, Lydian} (mode of the first melody)
- *mel_mode2* ∈ {Phrygian, Aeolian, Dorian, Mixolydian, Ionian, Lydian} (mode of the second melody)
- *exposure_grp* ∈ {Minimal, SDA, Lutheran, Non-musician, Musician}

We used three questionnaire responses—church attendance, village of residence, and church denomination—to assign participants to the three Uruwa exposure groups. As detailed in the Introduction, we can identify three major cohorts based on type and degree of exposure to Western or Western-like music in this region: those who attend SDA churches and hear or sing the SDA hymns (SDA group); those who attend Lutheran churches and hear or sing the stringben influenced hymns (Lutheran group); those who attend neither (minimal exposure group).

In the descriptive cadence model, there is a 4-way interaction between *cad_mode1*, *cad_mode2*, *tonic_pitch_diff_cat*, and *exposure_grp*. In the descriptive melody model, there is a 3-way interaction between *mel_mode1*, *mel_mode2*, and *exposure_grp*. We used a Student’s *t*-distributed prior with 7.763 degrees of freedom, a mean of 0, and a scale of 1.566 because this distribution on the logit scale approximates a uniform prior on the probability scale [59].

*Hypothesis-driven models*. For each of the hypothesis-driven models, two changes from the pre-registered versions (https://osf.io/qk4f9) were required. In the pre-registered models, there were high multicollinearities caused by interaction between the mean pitches of the two chords (or melodies), and model-fitting became unreliable. This interaction was simplified to a single term quantifying the difference in mean pitch between the two chords or melodies, which is actually the key quantity of interest and easier to interpret. Also, the data obtained from the questionnaire did not allow us to reliably distinguish between participants’ familiarity with Western or Western-like music and their familiarity with conventional Western associations between musical features and emotions (respectively termed *intr_fam* and *extr_fam* in the pre-registered models). In light of our experience with the region, the most sensible way to stratify exposure was on the basis of the questionnaire responses to church attendance, village of residence, and church denomination, as outlined above.

This led to the following *stimulus-level* predictors in the hypothesis-driven models:

- *cad_mode1* ∈ {Min, Maj} (type of the first cadence)
- *cad_mode2* ∈ {Min, Maj} (type of the second cadence)
- *diff_mean_pitch1_2* ∈ R (mean pitch difference between the two stimuli, in semitones)
- *timbre* ∈ {Strings, Voices}
- *melody* ∈ {‘1’, ‘2’ ‘3’} (the three melodic subjects—as notated in the S1 File—coded categorically).

In the cadence models, the principal predictors of interest are *diff_mean_pitch1_2* and the interaction of *cad_mode1* and *cad_mode2*. The *cad_mode1:cad_mode2* interaction represents the four different sequences of cadence types: major to major, minor to minor, major to minor, and minor to major. For the effect of cadence type we were, therefore, particularly interested in the contrast between the latter two. Including mean pitch difference in the cadence model means that any modelled effects of cadence type arise from aspects not correlated with mean pitch, such as the previously-mentioned psychoacoustic features of roughness, harmonicity, and spectral entropy. These three predictors interact with *timbre*. In the melody models, there is a 3-way interaction between *diff_mean_pitch1_2*, *timbre*, and *melody*. In the cadence and melodies models, there is just an interaction between *diff_mean_pitch1_2* and *timbre*.

In every hypothesis-driven model, all within-participant effects were modelled as varying (random) effects across participants. Weakly informative priors were used on all population-level effects: Student’s *t*-distribution with 3 degrees of freedom, a mean of 0, and a scale of 1. The use of varying effects and weakly informative priors reduces the probability of false positives and overfitting when data are noisy [60, 61].

A separate hypothesis-driven model was run for each of the five exposure groups rather than using a single model with an exposure group interaction; this allows for partial pooling of information between participants within each group, but not between groups (in an exposure interaction model, each group’s estimates would be drawn towards the overall mean due the varying effects and shared prior). Ensuring complete separation between groups is appropriate because one of the fundamental research questions is whether some of them (notably the minimal exposure group) may have very different responses to the others. The hypothesis-driven models’ formulas and full output summaries are provided in the S1 File.

## Results

The descriptive model, detailed in Methods, allows overall patterns in the data to be visualized (Fig 1) with cadences represented on the left and melodies on the right. This figure shows the probability of choosing the second stimulus as the happy one, with the colours representing the pitch difference between the first and second stimulus in each trial. In order to test the differing effects of cadence type and mean pitch, we tested the four hypotheses introduced earlier. Bayesian multilevel logistic regression was used to assess evidence both for and against these hypotheses, and to estimate their expected effect sizes (summarized in Table 1 and Fig 2). We assess evidence using evidence ratios, which are probability ratios in favour of directional hypotheses [62]. These are subsequently detailed in the main text along with any strongly evidenced moderating effects of timbre. As detailed in the Methods, a unit increase in any predictor represents an additive change on the logit scale assumed to underlie participants’ binary decisions. In addition, we also report results from ‘ROPE’ (Region of Practical Equivalence) tests, which tests the probability of an effect being practically equivalent to zero [27].

**H1—Cadence type: The effect of Maj to Min compared to Min to Maj, adjusting for mean pitch and timbre**. There is very strong evidence for a large positive effect of major cadences on self-reported happiness in both Sydney groups and the SDA group, and strong evidence in the Lutheran group. For the minimal exposure group, there is no convincing evidence for an effect in either direction; nonetheless, there is only a 23% probability this effect is practically equivalent to zero. Hence, for this group, a convincing claim cannot be made either for or against an effect of cadence type.

**H2—Cadence mean pitch: The effect of a 1-semitone increase in mean pitch, adjusting for cadence type and timbre**. There is very strong evidence for a large positive effect of mean pitch on self-reported happiness in both Sydney groups and strong evidence for a small effect in the Lutheran group. For the SDA group, however, there is evidence (a probability of 0.90) for there being practically no effect, while in the minimal exposure group there is no convincing evidence either for or against an effect. Regarding the moderating effect of timbre, for the Lutheran group, there is a strong evidence ratio (10.70) that the effect of mean pitch difference is greater for strings than for vocal timbres.

**H3—Melody mean pitch: The effect of a 1-semitone increase in mean pitch, adjusting for timbre and melodic subject**. There is very strong evidence for a large positive effect of mean pitch for both Sydney groups and a medium effect for the Lutheran group. There is weak evidence in favour of a small effect in the SDA group but, for the minimal exposure group, there is neither evidence for an effect nor for its practical absence.

**H4—Cadence and melody mean pitch: The effect of a 1-semitone increase in mean pitch, adjusting for timbre**. Combining the cadences and melodies data allows us to assess the effects of mean pitch from a greater variety and number of musical stimuli (although there is no longer any adjustment for cadence type or melodic subject). There is very strong evidence for a large positive effect of mean pitch for both Sydney groups, as well as smaller effects in the Lutheran and SDA groups. For the minimal exposure group, there is no convincing evidence in favour of an effect in either direction.

## Discussion

The results demonstrate that the self-reported emotional effects of major and minor—operationalized as cadence type and mean pitch—are strongly associated with exposure to Western or Western-like music. That said, for cadence type, we cannot rule out the possibility of a universal effect (resulting from the psychoacoustic properties discussed earlier) because the estimate from the minimal exposure group is insufficiently certain to confirm or deny. For mean pitch difference, the results in the minimal exposure and SDA groups point to only a small or practically non-existent effect. This suggests that the effect of mean pitch on valence—remarkably powerful in the West—is essentially cultural.

Given that none of the Uruwa groups are exposed to conventional (cultural) Western associations between musical features and non-musical emotive events in the Uruwa area, the effects of cadence type in those groups most likely arise from familiarity rather than from associative conditioning. Indeed, a supplementary analysis of twenty Lutheran hymns performed in the *stringben* style [44] by musicians from Towet (detailed in the S1 File) showed that all were in a major key, and minor chords were used for only 8% of the musical beats; all other chords were major. Supplementary analysis of harmony in the *SDA Hymnal* shows more variety than the Lutheran hymns, but it also comprises a preponderance of major chords (86%) over minor (12%) (detailed in the S1 File). This provides a ready explanation for the major cadences inducing more happiness than the minor—to participants in the SDA and Lutheran groups, they sounded more familiar.

The SDA hymns analyzed do not, on average, comprise more ascending sequences than descending: each piece almost always starts and ends with the tonic chord in a similar register, which implies the prevalence of upwards and downward motions of mean pitch must be approximately equal. Given the absence of associative conditioning, this could provide a natural explanation for why, in the SDA group, the effects for mean pitch are so small (and smaller than those for cadence type). However, it is not clear why this is not also the case for the Lutheran group, which exhibits a mean pitch effect more comparable to that of cadence type. A possible explanation would be that traditional music in this group differs from the others, but local knowledge suggests otherwise and our supplementary analysis shows no evidence that pitch difference signifies emotive valence in such music.

Across all of the stimuli, effects are stronger for the Sydney participants than for the Uruwa participants. This may be down to greater familiarity with Western music; it is likely also due to an additional influence of associative conditioning from conventional spatiotemporal associations of major with happy, and minor with sad—something we are sure is absent in Uruwa. The stronger effects for Sydney musicians compared to non-musicians may result from the former’s additional familiarity with musical structure and more firmly embedded associative conditioning, and also from them having better skills at discriminating the stimuli (some of which were quite similar) due to long-term explicit musical and ear training which is absent in the non-musicians as well as all the Uruwa groups.

The results should be interpreted with some caution, due to several limiting factors. First, participants may have a lack of familiarity with the notion of someone presenting stimuli multiple times and asking for a response or an opinion. This was encountered previously by Mulak et al., [46], who conducted a word learning study in the same area, and interpreted their null result as possibly reflecting a lack of familiarity with the task. Important to note is that Mulak et al., [46] concluded this based on a predominance of patterned responses, a type of response we already excluded from consideration in this study. Many research experiments are designed with a particular cultural influence and these designs may be interpreted differently in another culture [46, 63]. We attempted to design the experiment in such a way that it would be as meaningful as possible for the community members, but it remains a foreign task. If the opportunity arises to visit the area again, we would include measures to try and reduce the lack of familiarity as much as possible although it would be very difficult to design a task that is fully translatable across cultures. On a similar note, the notion of valence and the response options could be checked in a control task with clearly emotionally loaded stimuli (such as speech excerpts) to validate whether participants provide meaningful results; although even that precaution would not provide certainty the actual task is fully understood. Additionally, in order to obtain evidence for the absence of any meaningful effect in the minimal exposure group (or detect a rather small effect), we would need to collect more data.

## Conclusion

In sum, we find no evidence for a universal effect of major harmony or melody inducing greater reported happiness than minor. These results are similar to the majority of cross-cultural music affect studies discussed in the Introduction [18, 19, 24–26]. That said, our Bayesian approach also allows us to assert a lack of firm evidence against such a universality, which would not have been possible had we used frequentist statistics. We find convincing evidence that the induction of positive affect by major, compared to minor, is enhanced by exposure (familiarity) and by associative conditioning: effects are close to decisive for Sydney musicians and very strong for Sydney non-musicians, both of whom have considerable exposure to conventional pairings of musical features and emotions; they are smaller in the Lutheran and SDA groups, which have some exposure to Western or Western-like music but not its conventional associations; and smaller still in the group with minimal exposure to Western-like music. The apparent inconsistency between our results for the minimal exposure group and those reported by Fritz et al. [24] may be due to the additional tempo cues provided in the latter, which are not present in the current study.

The results reported here show the importance of conducting cross-cultural studies in communities with differing levels of familiarity with Western music, and its cultural context; it is only through such studies that we can uncover the commonalities and varieties at the heart of music cognition.

## Supporting information

S1 File Contains all the supporting tables and figures. (PDF)
