# Supplementary material for: Emotional responses in Papua New Guinea show negligible evidence for a universal effect of major versus minor music
Source: PLoS One. 2022 Jun 29;17(6):e0269597. doi: 10.1371/journal.pone.0269597 (PMC9242494; doi:10.1371/journal.pone.0269597)
Supplement: S1 File — (PDF) [file pone.0269597.s001.pdf]

Supplementary Information

A. Definitions of musicological terms

Western music. We define this term to mean any music that makes substantial use of a set of structural features commonly found in European music from at least the 17th century (although some of these date substantially further back in the European tradition; some, somewhat later). These include the use of:

- instruments (and human voice) that produce harmonic complex tones and, hence, induce clearly perceptible pitches;
- a set of discrete pitches tuned to a meantone-like system, the most common of which, in contemporary practice, is 12-tone equal temperament where every octave is divided into 12 equal semitones (a meantone system contains intervals (octaves) with a frequency ratio close to 2/1, and intervals (perfect fifths) with frequency ratios close to, or slightly smaller than, 3/2, which ensures that 4 perfect fifths minus 2 octaves approximates a frequency ratio of 5/4);
- frequent use of the diatonic scale (a *well-formed scale* [1] with 5 large steps and 2 small, where the large steps are approximately twice the size of the small)
- frequent use of major and minor chords and, sometimes, diminished and augmented chords, and their extensions (sevenths, ninths, etc.);
- *modulations* between diatonic scales, which are typically smooth because the two scales will share many common pitches and are typically mediated via a pivot chord that is common to both scales;
- common assertion of a tonic pitch class or tonic major or minor chord through the use of cadences, which are well-established chord progressions that typically involve movement from a dominant seventh chord (or major chord) to a major or minor chord a perfect fifth below and, often, this dominant chord is preceded by a chord containing the scale's fourth degree (the subdominant);
- there is an isochronous hierarchical binary or ternary metrical structure, whereby the fastest metrical level (rhythmic pulse) is grouped into either twos or threes to make a slower metrical level, which is itself grouped into twos or threes to make an even slower metrical level, and so on.

This definition of Western music is, therefore, one that allows for Western music or *Western-like music* to be produced in non-Western countries. For example, the Western-like guitar band music in PNG is strongly informed by Western music (through historical musical training provided by missionaries and the use of Western musical instruments[2]) and comprises almost all of the characteristics of Western music (as defined above), whilst still being quite distinct and recognisable as a genre or style of music that is different from anything actually produced in the West. Of course, this definition of Western music may seem flawed. For example, twentieth century atonal (including most serial) music would not fulfill all of the above criteria and, yet, is clearly a Western phenomenon. However, it is the term 'Western music' that is problematic here, rather than the definition provided above; unfortunately, no other English term is available that can capture all and only the set of features above; furthermore, we feel that in most readers' minds 'Western music' will most readily evoke the characteristics listed above, and this motivates our choice of a practical, though imperfect, term.

Major and minor. Common definitions vary depending on whether they are applied to harmony (individual chords or successions of chords) or to melody (successions of pitches). Here, we omit a few definitional nuances so as to concisely summarize them. Major and minor chords both comprise three distinct *pitch classes* (pitch classes consider any two pitches an octave apart to be the same and are expressed in semitone units). Relative to the chord's *root*, the pitch classes of major and minor chords are, respectively, $(0,4,7)$ and $(0,3,7)$. Hence the mean pitch of a major chord is higher than that of its analogous minor chord.

For sequences of chords, major and minor typically refer to the tonic chord (the *tonic* is the chord or pitch class that serves as a theoretical 'reference', or musical 'centre' or 'home' of the sequence) or to the preponderance of chords in the sequence. In the experiment, we use cadences comprising mostly major chords ending on a major tonic, and cadences comprising mostly minor chords ending on a minor tonic. In general music theory, cadences often indicate the end of a musical phrase [3].

A melody is a sequence of pitches which, like a chord, can be summarized as pitch classes. If the melody has a pitch class 4 semitones above its tonic, it is typically deemed major; if, instead, it has a pitch class 3 semitones above the tonic, it is typically deemed minor. So this definition is analogous to that for chords. However, a more nuanced definition takes into account the intervals between every pitch class in the melody and its tonic. In this experiment, we make use of melodies from 6 (out of a possible 7) *modes* of the *diatonic scale*. The diatonic scale is a well-formed pattern of 5 tones (large steps) and 2 semitones (small steps); each mode of the diatonic scale simply defines which of those scale degrees is considered the tonic. In practice, a piece of music in a given mode will typically start and end on its tonic or otherwise emphasise it; for example, using it as a drone.

The seven different diatonic modes can be ordered by their mean pitch relative to their tonic: Locrian (not traditionally used in Western music, nor in the experiment), Phrygian, Æolian (the 'natural' minor scale), Dorian, Mixolydian, Ionian (the major scale), and Lydian. By this ordering, Phrygian can be considered the most minor mode in the experiment; Lydian the most major. The first three modes have a pitch class that is 3 (but not 4) semitones above the tonic; the latter three have a pitch class that is 4 (but not 3) semitones above the tonic.

B. Background information on the Uruwa River valley

General background. The Uruwa River Valley is a remote twelve-village area in the Saruwaged Mountains, Morobe Province, Papua New Guinea. A map of the Uruwa River valley with its villages is presented in Figure 1. Elevation in the area and the surrounding mountains ranges from sea level to peaks of over 4,000 m. As described by Sarvasy[4], there is hardly any level ground and people live, move and cultivate their crops on steep slopes.

As is common in such remote mountain areas of PNG, there are no roads to the area or nearby mountain regions. The Uruwa River Valley is accessible to outsiders only by small airplane; in the six-village southern, higher-elevation part of the river valley, such airplanes must land on an inclined grassy airstrip at Yawan village that was cleared by villagers, using hand tools, over several years in the 1970s, then extended in the 1990s. Historically, the village communities of the Uruwa River Valley are said to have lived in a state of uneasy truce with each other, punctuated by conflicts [5]. This is reflected in the locations of the villages–each is separated from the others by geographic barriers, such as waterways. Each village community comprises two or more clan groups.

Musical background. There are two main strands of musical traditions in the Uruwa region, as elsewhere in the region [6]. Older traditional genres are accompanied by hourglass-shaped hand-drums (called *uwing* in Nungon) or by flute. Since the 1970s, a style of Western-influenced sung genre, called *stringben* in Tok Pisin (from *string band*), and characterized by guitar or ukulele accompaniment, has co-existed with the older musical styles [7, 8]. Music is primarily heard in weekly church services, on special occasions, or when individuals sing while going about daily activities, or practice for performances. There are no professional musicians, nor people who specialize in musical performance; traditionally, all women sang and danced in communal gatherings, and all men sang, danced, and played *uwing*. Elementary-age children may learn to sing songs in the local language, Nungon, or in Tok Pisin, at school. Few people in the area own radios or other music players, and there is no mains electricity source for charging mobile electronic devices. There is no equipment for viewing movies or videos in the area; nor, for that matter, are any of the Uruwa people who live in distant diaspora areas known to own televisions.

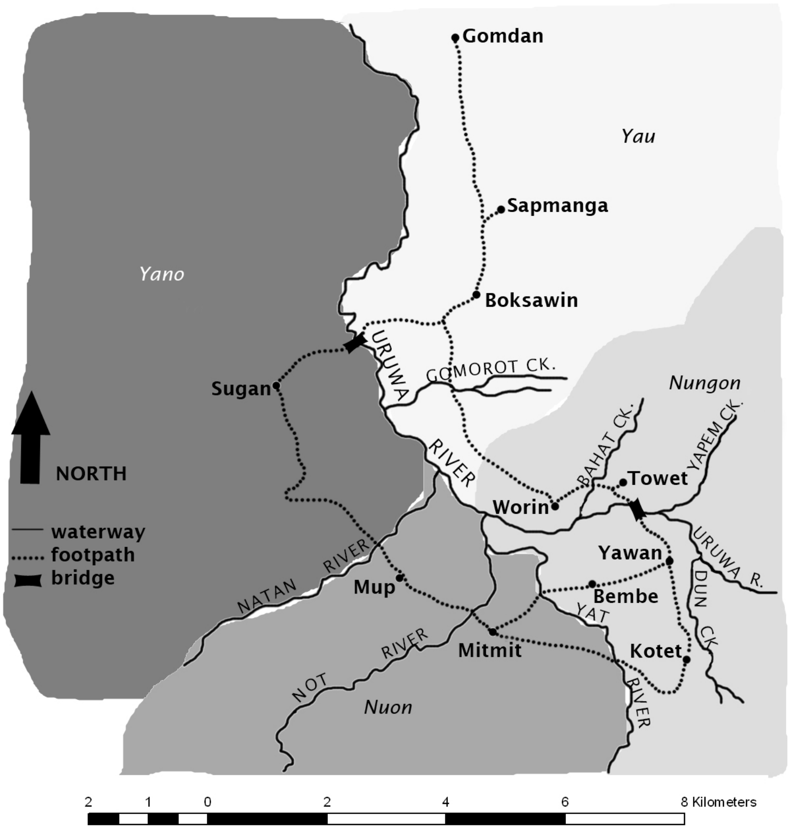

Fig. 1. Map of the Uruwa River Valley with villages (bold), rivers (capitals), and language areas (italics). Shadings represent mid-level dialect groupings.

Linguistic background. Linguistically, each village had its own language variety, with distinctive pronunciation and vocabulary. One historical dialect, that of Mitmit and Bembe, is nearly obsolete today, due to mass deaths, then intermarriage, several decades ago [4]. Although the term *Nungon* is used nowadays to describe the entire dialect continuum of the six-village, southern Uruwa River Valley communities, this term simply means 'what', such that 'I speak Nungon' can be understood to mean 'I say *nungon* for 'what'', as opposed to the people of the northern Uruwa villages, who say *yao* or *yano*. As seen in the shading in Figure 1, the term for 'what' is actually *nuon* in the dialects of two villages of the southern six (Mup and Mitmit), but structurally these dialects are more similar to those of the four other villages (Worin, Towet, Yawan, and Kotet) than to the northern Uruwa varieties, so these are considered part of the major grouping labeled Nungon. Judging by historical records from 20th-century linguistic surveys of the region [9], it is more traditional, and even today, more precise, to refer to the speech varieties by the village name: so the Towet dialect, Worin dialect, and so forth. The languages of the Uruwa area are classified as belonging to the Finisterre branch of the Finisterre-Huon family of Papuan languages, which straddles the Huon Peninsula and runs into the Finisterre Range, along the border of Madang and Morobe Provinces.

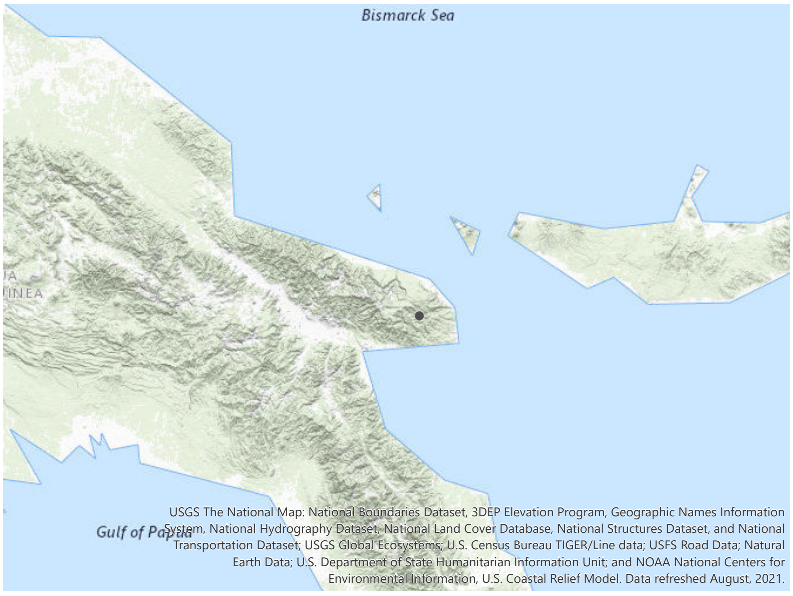

Fig. 2. A map of the Huon Peninsula. Image from the USGS National Map viewer (http://viewer.nationalmap.gov/viewer/)

Historical background. The first European visitor to the region, the Swiss missionary Saueracker, passed through the Uruwa area in 1928 from the region to the east of the Uruwa area, where the distantly related Nukna language is spoken. But most early missionary activity in the Uruwa River Valley was done by Papua New Guinean missionaries who lived in the region for many years, and also introduced coffee farming, cabbage, pumpkin, peanuts, and some other crops. By the 1960s, most people in the area had been baptized as Lutheran Christians. The Lutheran church used another Papuan language, Kâte, as a lingua franca in much of northeast New Guinea, and some older Nungon speakers attended a Kâte school and became literate in Kâte. Songs in the Kâte language are still known by Nungon speakers, and performed occasionally in the Lutheran churches in Worin and Mup. The Lutheran church was known for welcoming local musical traditions and encouraging the use of *uwing* drums and local languages in services. With the advent of the *stringben* style, that also became incorporated into Lutheran services. In contrast, the later-arriving Seventh-Day Adventist church, which made inroads in the Uruwa area from the late 1970s on, strictly prohibited use of any PNG music styles in church services: hymns must be drawn from the official *SDA Hymnal*, and sung to traditional North American and European melodies. People baptized into the SDA church had to renounce playing the *uwing* drum, in addition to the major lifestyle shifts required of SDA adherents: abstention from consuming pork (traditionally, central to feasts and gatherings in much of PNG), tobacco and betelnut. Today, Towet village is the only Uruwa village in which the majority of people adhere to the SDA church. In all other villages, SDA followers are either in the minority or non-existent. Lutheran churches today are found in Worin and Mup villages,

and SDA churches are found in Towet, Yawan, Kotet, and Worin villages. There used to be a Lutheran church in Kotet, which was demolished at the end of 2011.

Economically, the Uruwa area was traditionally linked to other parts of the Huon Peninsula (northeast PNG) by the Vitiaz Strait trade circuits and other trade circuits, whereby Uruwa forest products were traded for coastal products such as clay vessels (see Figure 2). This tradition continued through the 1990s and many Uruwa families maintain "trade-friend" relationships with families at centers along the traditional trade circuits, and have working understanding of the languages spoken in their regions.

A major cultural shift began in 1995 in the southern Uruwa villages, when Towet man Dono Ögate and his wife Eni, who had married into the area from the Nukna region to the east, returned to the region from the port city of Lae and began a concerted effort to 'develop' their community. Eni trained as the founding teacher of the first elementary school in Yawan village, established in 1998, and together the couple began distributing non-traditional clothing, such as T-shirts and shorts, to their community, and teaching them to speak and read the English-based creole Tok Pisin. In 2019, Dono Ögate was recognized for this work by the Digicel Foundation: he received the national 2019 Overall Man of Honour award.

In 2009, the YUS Conservation Area (encompassing much of the Uruwa villagers' highest-elevation landholdings and extending into the neighbouring Som and Yupna regions) was established, through the efforts of local people and an organization, the Tree Kangaroo Conservation Program (TKCP), linked to Seattle's Woodland Park Zoo. Since then, TKCP has been the major instigator of small-scale development and aid initiatives in the region, and employs a handful of people throughout the YUS Conservation Area, who travel regularly between the TKCP office in Lae and their home villages. The YUS Conservation Area also attracted other academic researchers to the area, including teams from James Cook University (JCU), Conservation International (CI), and the New Guinea Binatang Research Centre (BRC). Most of this research was biology-focused, with the exception of some engagement with villagers around livelihoods (JCU), and the long-term linguistic research by H.S.S.

The majority of this research brought only short-term employment opportunities for a handful of local people, spanning either the length of the foreigners' field trip, or at most 1–2 years (in the case of BRC). In perhaps the longest-running contractual scheme, H.S.S. has paid several local people, including the three organizers of the 2019 Towet "research fair" to which these experiments belonged, to record and transcribe child speech in Nungon since 2015, as part of a long-term longitudinal study of child language development in the region.

Whether the events described in this subsection significantly impacted participants' musical experiences over time is uncertain (indeed, models with participants' age as a smooth spline effect, which 'interacted' with our principal effects, did not give consistent or decisive results) but they provide a perspective on changes happening in the area. A more thorough description of the history of the Uruwa River valley can be found in Sarvasy[4].

C. Analysis of traditional music recordings from Kotet, Yawan, Towet, and Worin

In order to gain some insight into the traditional music across across the area, we analysed six songs from a larger set of audio recordings made by H.S.S. in Kotet, Yawan, Towet, and Worin in 2011–13. The songs were (with a short description):

- **Ex. 1** Joyous song for decorating a young initiand. Performed by Manggirai, a man in his 60s. From Kotet.
- **Ex. 2** Melancholic song sung to the singer by her deceased daughter in a dream. Performed by Inewe, a woman in her 60s. From Kotet.
- **Ex. 3** Melancholic traditional song associated with a legend. When out on a hunting trip with her sister and brother-in-law, a woman enters a cave and has her head chopped off by a demon. Her sister finds her, returns her head to her neck with sticky sap, and hangs the handle of a heavy string bag of game from her forehead, to hold her head in place. Then, as they make their way down the mountain to meet the brother-in-law, the sister sings a mournful song bemoaning her sister's accident. Performed by Fooyu, a woman in her 50s. From Yawan.
- **Ex. 4** Traditional song associated with preparing to slaughter a pig for a feast: joyous. Performed by Nongi, a man in his 70s. From Towet.
- **Ex. 5** Song composed by the late husband of the singer, as he gazed out over their landholdings and prepared to die. Performed by Irising, a woman in her 60s. From Towet.
- **Ex. 6** Exemplar of the songs that young men sing from a ridge above the village on returning from a successful hunt, alerting their relatives below to start cooking vegetables to accompany the meat. Performed by Yamosi, a boy in his late teens. From Worin.

In all but one recording, there is just one singer. In Ex. 3, a second singer follows – with a small lag – the main singer's pitch in unison (approximately the same pitch). As shown through the notes above, we selected one song with positive valence and one with sad valence from each village, where available. For Yawan, we chose one sad song because no happy songs were recorded (other recorded songs are in a 'spirit language' and are not obviously interpretable as positively or negatively valenced); for Worin, we chose one happy song because no sad songs were recorded.

The pitch intervals in the songs are frequently noticeably microtonal (by which we mean they do not necessarily correspond to the intervals of Western music); furthermore, the pitches are often rather inconsistent. For these reasons, it would be inappropriate for the analysis to rely on a Western notational system of pitches; instead, we use computational pitch detection with fine resolution across both pitch and time. The pitch detection was performed with MATLAB's Audio Toolbox using the normalized correlation function [10, 11] in 52 ms windows with 42 ms overlaps. For each window, the harmonic ratio (periodicity) of the audio was also calculated in order to select only those portions of the detected pitches that correspond to clearly pitched sounds (such as sung vowels). For each song, the threshold at which the harmonic ratio was deemed high enough to include that portion of the detected pitch, and the pitch range over which the detection algorithm searched, were adjusted by hand to produce a comprehensive but clean pitch envelope.

In Figure 3, for each of the six songs, we graph: (1) on the left, the vocal pitch envelope over the song's duration and its mean pitch (dotted line); (2) in the middle, a smoothed density plot of pitches over the song's entire duration (this is a nonperiodic absolute monad expectation vector); (3) on the right, a smoothed density plot of all pitch intervals in the song (this is a nonperiodic relative dyad expectation vector, which is similar to an autocorrelation of the pitch density but, crucially, each individual pitch does not contribute any density at an interval of zero size) [12]. The graphs for (2) and (3) were calculated using the `expectationTensor` function from the Music Perception Toolbox (https://github.com/andymilne/Music-Perception-Toolbox); the MATLAB script calling this function, and the pitch detection functions, is available at https://osf.io/qk4f9.

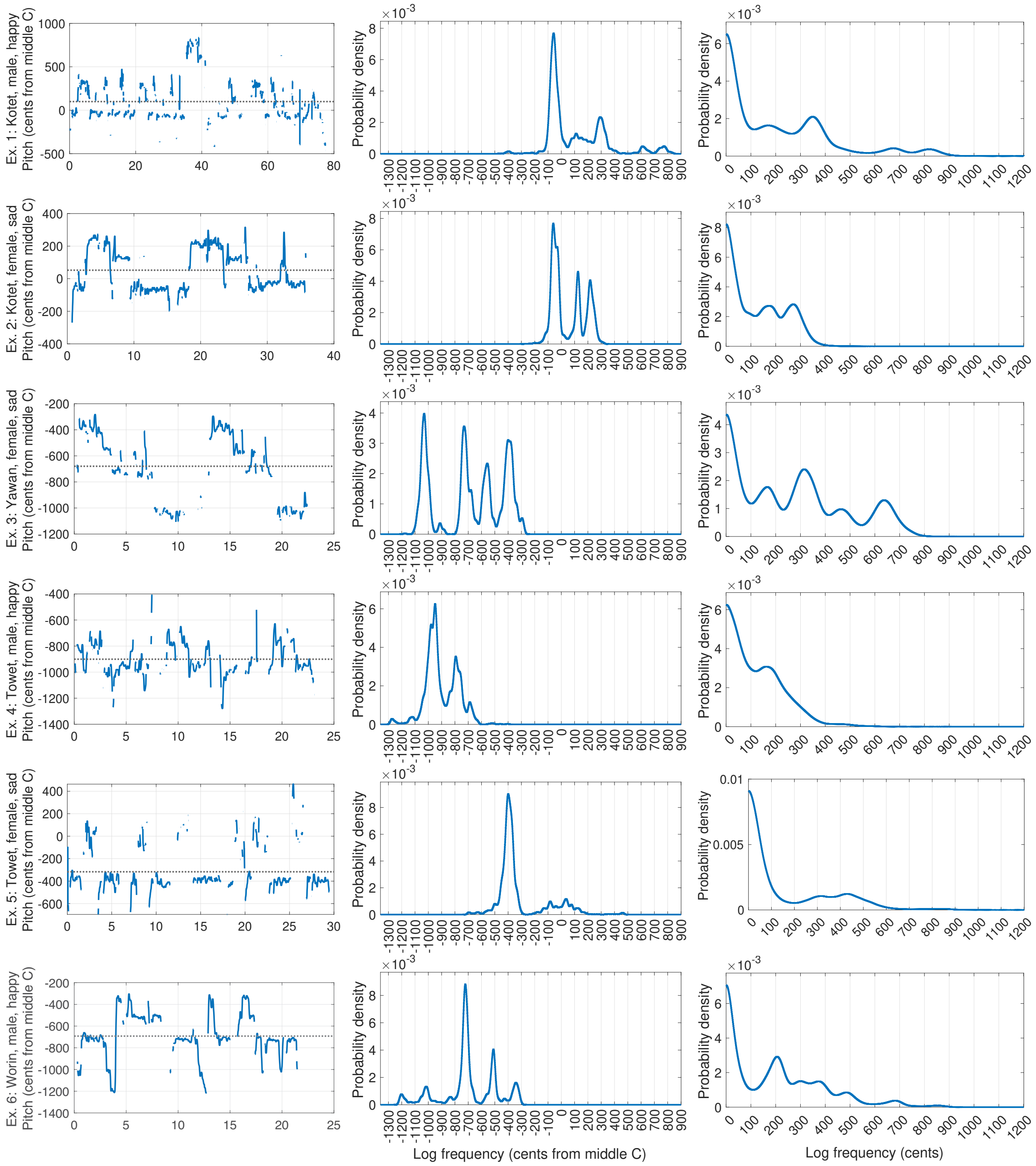

Fig. 3. For six songs: pitch envelope (left) in seconds; pitch density (middle); interval density (right).

Informed both by listening and the computations summarized in Figure 3, the pitch content of each piece is now briefly outlined (cents are 100th of standard semitone hence 1200th of a standard octave):

Ex. 1 (Kotet, male, happy) comprises more than 6 distinct pitches and is the most complex song to analyse, in part due to a gradual downward shift of the *focal* (most common) pitch, which gradually drops by about one third of a semitone over the first half of the song. The next most frequent pitch is approximately 350 cents higher than the focal pitch, often connected to it by one or more intermediate pitches which are rather variable in tuning. There are also occasional lower pitches, which decorate the focal pitch; these are also variable in tuning. Strikingly, two substantially higher pitches occur midway through the song, which are approximately 7 and 8.5 semitones higher than the focal pitch.

Ex. 2 (Kotet, female, sad) comprises 3, or possibly 4, distinct pitches. There is a focal pitch midway between B3 and C4 and two higher pitches, which are approximately 2 and 3 semitones higher. The final two ‘reiterations’ of the focal pitch, however, are sung about 40 cents higher than before creating, for my (A.J.M.) ears, the possibility of a distinct new pitch.

Ex. 3 (Yawan, female, sad) comprises a repeated descending sequence of 4 distinct pitches, which loosely approximates a narrowed version of the familiar pentatonic pitch class subset $(0, 3, 5, 7)$ (the outer interval is actually more like 6.5 semitones). This song does not exhibit a unique focal pitch that is emphasised substantially more than any other.

Ex. 4 (Towet, male, happy) has 4 distinct pitches: the song alternates between principal pitches approximating D3 and E3 (of which the first is focal), which are decorated with pitches approximating C3 and F3.

Ex. 5 (Towet, female, sad) has, perhaps, 3 distinct pitches. There is a distinct lower focal pitch at A♭3. The is another higher pitch, which is rather flexible in tuning and often performed with pitch swoops centred approximately a major third higher than the focal pitch. Near the end there is a brief instance of a distinctly higher pitch about 8 semitones higher than the focal pitch; hence the three pitches loosely approximate those of an augmented triad.

Ex. 6 (Worin, male, happy) has 5 distinct pitches approximating C3, D3, F3, G3, A3 and has a distinctly anhemitonic pentatonic flavour with pitch intervals closely approximating those found in Western music. The focal pitch is F3.

We can pick out some interesting generalities across these songs. The mean pitch sung by male and female singers is similar: respectively, 315 and 498 cents below middle C, which are approximately A3 and G3, respectively. The females, therefore, are singing in what would be considered, in Western practice, to be a fairly low register (although close to the typical fundamental frequency of female speech). The pitch range covered by the principal pitches rarely exceeds a perfect fifth (although small in comparison to Western art music, ranges such as this are common in European folk songs). The number of distinct pitches is relatively small (although, given their variability, it is not always clear which pitches are distinct). There is often a focal pitch (or small range of pitches) that is sung substantially more than any other pitch; the only clear exception is Ex. 3, where no pitch seems clearly favoured. The pitches are often sung with noticeable variation – this can be seen in the central column of plots where the peaks are relatively wide.

In terms of intervals between pitches, as shown in the right column of plots (note that these are intervals across the entire song and so include intervals between pitches widely separated in time), we see a clear preponderance of very small intervals. These result from approximate continuations of, or close repetitions of, some pitches (i.e., the broad peaks in the pitch density plots). Beyond that, there is common use of an interval that is a 'small' whole tone (180–200 cents), as well as a variety of larger intervals that are often inconsistent within, and certainly between, performances. These larger intervals range up to a maximum of about 850 cents but, as mentioned above, are uncommon greater than about 700 cents. It is worth noting that – beyond the unison interval of 0 cents – there is little correspondence to melodic intervals common in Western music: peaks in the right column of plots typically fall between the semitone grid lines. An exception is Ex. 6, where there are peaks close to all semitone intervals between 200 and 700 cents.

In terms of tonal structure, it is plausible that the focal pitch in a song acts as something loosely analogous to a tonic: a point of frequent return against which the other pitches are intended to be mentally compared. It is worth noting that, of the examples with a distinct focal pitch (1, 2, 4, 5, and 6), the focal pitch is the lowest of the principal pitches in every example except the sixth. Every song's mean pitch minus its highest density pitch is, in order of example number, 158, 113, 352, 48, 83, and 30 cents.

With regard to whether the songs have happy (Ex. 1, 4, and 6) or sad (Ex. 2, 3, 5) words or performative contexts, there is no obvious association with the just described mean–focal pitch values; there is also no evident association with the number of distinct pitches, the inconsistency (spread) of pitches, or the overall range of pitches used. This accords with the way that Uruwa community members tend to talk about music; in ten years of association with the village, H.S.S. has never heard people describe the melodies of songs as evoking emotions alone; rather, the spare lyrics are the main conduit for emotion. Further, song lyrics themselves can involve the juxtaposition of imagery representing death, for instance, and new life. It is possible that more mournful songs that were not accompanied by dancing (such as Ex. 2, 3, 5) are traditionally sung with slower tempi than more joyous songs. There is also no obvious difference between the songs by village; an observation supported by Dono Ögate who stated that, for old music in Kotet, Yawan, Towet, Worin, Bembe, and Mup, 'individual songs are different across the communities, but the style is the same' (translated from Nungon by H.S.S.).

Of course, it may be that emotion is signified via the temporal ordering of pitches, such as might be captured by their contour, the tempo, and the vocal delivery and timbre. We do not focus on these temporal and performative aspects in this subsection because the principal features investigated in the main experiment (cadence type and mean pitch difference) are aggregated over time. As demonstrated in that experiment, in Western music, aggregated pitch features such as these are strongly related to emotive valence.

In summary, across the set of traditional songs analysed here, melodies are monophonic (sung solo or in unison) and rarely exceed a perfect fifth in range. There is typically a 'focal' pitch, which is sung more than any other. With the possible exception of a small whole tone of about 1.75 Western semitones, interval sizes are not consistent between songs and singers, and are somewhat inconsistent within each performance. They do not typically conform with Western intervals. There there is no evidence that the aggregated pitch content of each song (e.g., overall pitch range, number of pitches, or mean pitch relative to focal pitch) is associated with whether it has words or performative contexts that are either happy or sad; nor is it associated with the village of origin.

D. Analysis of *stringben* Lutheran hymn recordings from Towet

Thirty hymns were performed by a group of Towet musicians in two separate sessions: 1 male ukulele player and singer, 1 or 2 (depending on the session) male acoustic guitar players and singers, 1 boy *uwing* drum player, 2 or 5 (depending on the session) female singers also clapping. These were recorded by our research assistants in Worin in May 2020. Twenty of these were randomly selected and their chords analysed by ear. Every piece was in the key of E major (every piece started and ended with an E major chord and used standard cadences); for this reason, the table below shows the proportion of chords with Roman numerals relative to the tonic E major. Only four different chords were used across the twenty songs: the tonic (I) E major was the most common (it was played on 57% of beats); the dominant triad (V) B major was the next most common (21% of beats); the subdominant triad (IV) A major was the third most common (13% of beats); the least common chord was the submediant (VI) C♯ minor (8%). The E major chord was typically decorated with a thirteenth (C♯) (also known as an added sixth) at the start and end of each song (but not elsewhere), and the E major chord was typically decorated with a seventh (D) – making this a dominant seventh (V7) chord – only when it was the penultimate chord leading to the final tonic E.

Table 1. Chords used in twenty hymns performed by Towet musicians in the guitar band style. BPM gives the tempo of the song (the number of beats per minute), No. beats is the total number of beats in each song, the remaining columns give the numbers of beats occupied for the four chords played (I, IV, V, and VI) ignoring extensions (thirteenths and sevenths). The final row gives the median BPM and No. beats, and the percentages for each chord across all twenty hymns. Note that I, IV, and V are major chords, while VI is minor; hence 92% of chords are major and 8% are minor.

BPM	Key	No. beats	No. I beats	No. IV beats	No. V beats	No. VI beats
110	E maj	299	194	0	37	68
106	E maj	281	204	0	49	28
111	E maj	365	252	80	33	0
107	E maj	345	144	20	157	24
110	E maj	265	168	24	41	32
108	E maj	327	146	28	105	48
108	E maj	249	136	52	21	40
104	E maj	317	160	64	61	32
115	E maj	439	288	48	99	4
114	E maj	357	209	76	40	32
107	E maj	357	133	56	144	24
90	E maj	262	134	40	64	24
108	E maj	385	237	64	48	0
111	E maj	485	225	60	176	24
111	E maj	389	233	22	40	94
113	E maj	245	185	18	42	0
115	E maj	313	201	44	36	32
118	E maj	365	189	32	108	36
112	E maj	441	263	134	44	0
110		345	57%	13%	21%	8%

E. Analysis of SDA hymns

Thirty-one hymns were randomly selected from the *SDA Hymnal*, which is the source book for all hymns sung at all SDA services in the Uruwa area. They are presented in four-part harmony and were analysed to determine the proportion of chord types (major, minor, diminished, augmented, and suspended) in each hymn and across all hymns. Dominant 7ths and other extensions of major chords are categorized as 'major'; half-diminished and diminished 7ths as 'diminished', extensions of augmented chords are categorized as 'augmented'; suspended chords are categorized as 'suspended' only when they are not obviously passing to a major or minor chord (in which case they are categorized as major or minor, respectively; but these are exceedingly rare, anyway). The results are summarised in Table 2. In summary, there is more variety than found in the Worin recordings but there is still a substantial majority (86%) of major chords. The words for the two minor-key hymns are not intrinsically sad in nature; like most of the hymns, they are focused on praise and worship and have, if anything, a positive emotional valence.

Table 2. Proportions of chord-types over 31 hymns randomly selected from the *SDA Hymnal*. For each hymn, the proportions are the number of beats (quavers or crotchets; whichever is appropriate for the song) each chord type is played divided by the total number of beats with any chord in that hymn. Of the 31 hymns, only two are in a minor key. Averaging these proportions across all hymns, 86% of chords are major, 12% are minor, 1% are diminished; less than 0.5% are augmented or suspended.

Hymn name	Hymn No.	Key	Maj	Min	Dim	Aug	Sus4
There Is a Fountain	336	Bb maj	100%	0%	0%	0%	0%
Lord, Speak to Me	541	G maj	88%	9%	0%	3%	0%
Christ for the World	370	F maj	96%	4%	0%	0%	0%
Near, Still Nearer	301	Db maj	93%	7%	0%	0%	0%
Jesus Is All the World to Me	185	G maj	86%	14%	0%	0%	0%
Power in the Blood	294	F maj	100%	0%	0%	0%	0%
What a Friend We Have in Jesus	499	F maj	99%	0%	1%	0%	0%
When I Survey the Wondrous Cross	154	F maj	83%	17%	0%	0%	0%
Now Thank We All Our God	559	Eb maj	77%	20%	3%	0%	0%
Where Cross the Crowded Ways of Life	355	Ab maj	75%	19%	6%	0%	0%
Abide With Me	50	Eb maj	77%	22%	2%	0%	0%
My Maker and My King	15	C maj	94%	6%	0%	0%	0%
Glorious Things of Thee Are Spoken	423	Eb maj	89%	8%	3%	0%	0%
This Is My Father's World	92	D maj	92%	8%	0%	0%	0%
He Leadeth Me	537	C maj	91%	9%	0%	0%	0%
O Sing a New Song to the Lord	19	G maj	81%	19%	0%	0%	0%
Lead On, O King Eternal	619	C maj	85%	15%	0%	0%	0%
Ye Servants of God	256	G maj	85%	15%	0%	0%	0%
Just as I Am	314	D maj	100%	0%	0%	0%	0%
O Zion, Haste	365	Ab maj	88%	10%	0%	2%	0%
Now Let Us From This Table Rise	404	C maj	67%	29%	4%	0%	0%
Praise God, From Whom All Blessings	695	C maj	76%	24%	0%	0%	0%
I Surrender All	309	D maj	100%	0%	0%	0%	0%
Dear Lord and Father	481	C maj	86%	5%	5%	0%	4%
Shall We Gather at the River	432	Db maj	100%	0%	0%	0%	0%
God Has Spoken by His Prophets	413	F min	44%	48%	5%	0%	3%
O Love That Wilt Not Let Me Go	76	G maj	90%	10%	0%	0%	0%
Bless Thou the Gifts	686	G maj	88%	9%	0%	3%	0%
The God of Abraham Praise	11	E min	68%	30%	2%	0%	0%
I Will Follow Thee	623	Ab maj	99%	0%	1%	0%	0%
Give of Your Best to the Master	572	Eb maj	66%	28%	6%	0%	0%
			86%	12%	1%	0%	0%

F. Stimuli

Cadences

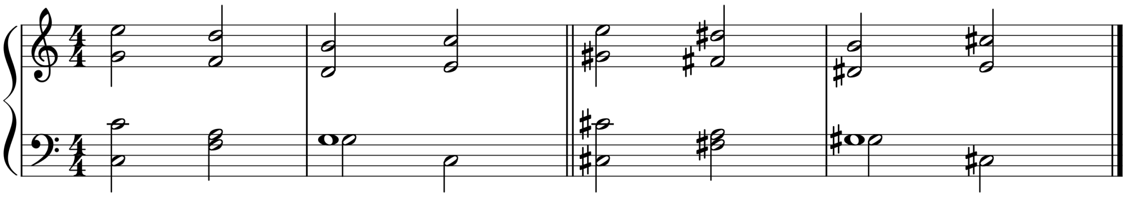

Fig. 4. Cadence example C major – C♯ minor. Each cadence consisted of either mostly major chords or mostly minor chords in order to make any effect of mode as clear as possible, and all chords in a given cadence were from a single diatonic scale in order to avoid any additional effects resulting from scale structure. All cadences had a tonic pitch of B, C or C♯ in order to decorrelate average pitch height from mode (hence allowing their separate effects to be disambiguated). Given there are 6 different keys (i.e., 2 modes $\times$ 3 tonic pitches), this would lead to a total of 30 ordered pairs (without repetition) to test, however for the sake of experiment time, it was decided to reduce this to a smaller number of pairs. The precise pairs of cadence keys were chosen to ensure that, for each participant, each cadence (characterized by its tonic and its mode) was heard an equal number of times (4 times out of 12 trials), that there was an equal split of major and minor cadences (6 each), and that the average pitch change across the 12 was zero. This led to two slightly different sets of 12 cadences which were allotted evenly between participants. Each cadence followed – as much as feasible within the above constraints – standard musical rules (e.g., voice-leading rules and chord choices). The only traditional compositional rule broken was the use of a minor dominant chord for the minor cadences in order to fulfil the first two constraints stated above.

Melodies

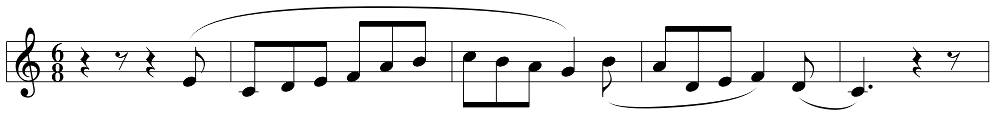

(a) Melodic subject 1

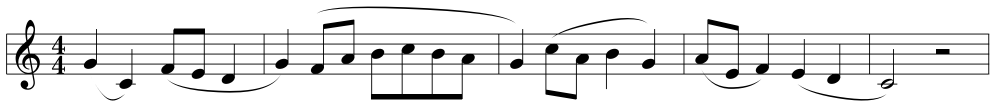

(b) Melodic subject 2.

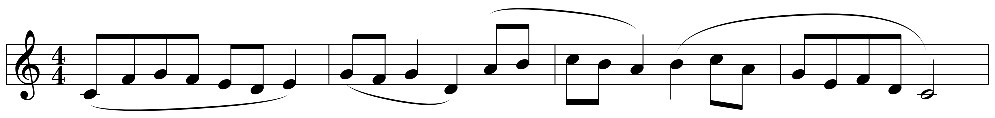

(c) Melodic subject 3.

Fig. 5. The three melodic subjects used in the experiments. Each subject was played in 6 different modes, obtained by changing the notated key signature: 1 sharp for Lydian, no accidentals for Ionian, 1 flat for Mixolydian, 2 flats for Dorian, 3 flats for Æolian, and 4 flats for Phrygian. All melodies were accompanied by a quiet note on C2 and C3. All notes were played legato except for the notes at the end of each slur, which were slightly shortened to help separate the musical phrases. Ignoring these subtle phrase-motivated variations in durations, within each melodic subject, the notes whose pitches changed between any two modes (D, E, F, A, and B) had the same overall duration; for example, in the first subject, every such pitch sounds for a combined duration of 3 quavers (e.g., three quavers or one crotchet and one quaver).

G. Participant demographics and data exclusions

Table 3. Test locations and participant numbers

Dates	Test location	Participants' origin	No. Participants	Experimenters
27 June–7 July 2019	Towet	Towet	87	E. A. Smit, A. J. Milne
27 June–7 July 2019	Towet	Worin	1	E. A. Smit, A. J. Milne
14–25 July 2019	Mup	Mup	18	B. Waum, N. Urung
14–25 July 2019	Mup	NA (missing interview)	1	B. Waum, N. Urung
30 July–4 August 2019	Mitmit	Mitmit	1	B. Waum, N. Urung, N. Ögate
30 July–4 August 2019	Mitmit	Bembe	19	B. Waum, N. Urung, N. Ögate
30 July–4 August 2019	Mitmit	Worin	3	B. Waum, N. Urung, N. Ögate
6–8 August 2019	Kotet	Kotet	15	B. Waum, N. Urung, N. Ögate
1–3 October 2019	Kotet	Kotet	16	B. Waum, N. Urung, N. Ögate
3–8 October 2019	Yawan	Yawan	9	B. Waum, N. Urung, N. Ögate

Table 4. Overview of numbers of blocks before and after exclusions due to patterned responses.

Group	Block	No. blocks	
		Before Exclusions	After Exclusions
Uruwa: all	Cadences	169	111
	Melodies	169	122
Uruwa: minimal	Cadences	29	17
	Melodies	29	19
Uruwa: Lutheran	Cadences	44	35
	Melodies	44	36
Uruwa: SDA	Cadences	96	59
	Melodies	96	67
Sydney: all	Cadences	79	78
	Melodies	79	79
Sydney: non-musician	Cadences	60	59
	Melodies	60	60
Sydney: musician	Cadences	19	19
	Melodies	19	19

Table 5. Nationalities of the Sydney participant group

Nationality	No. participants
Australian	41
Bangladeshi	1
Chinese	7
Egyptian	1
Greek	4
Indonesian	3
Iraqi	4
Lebanese	6
Middle Eastern (other)	1
New Zealand	1
Pakistani	1
Persian	1
Russian	1
Spanish	2
Sudanese	1
Tibetan	1
Vietnamese	1
Not provided	2

H. Ethics and risk management considerations

Ethics We acknowledge that conducting research cross-culturally is sensitive and requires extensive work to ensure a fruitful collaboration for all parties involved [13]. We appreciate the many challenges involved in cross-cultural research and we aim to be transparent about the processes involved in the current study. Here, we outline the steps that were undertaken to ensure that the presence of researchers would not adversely impact the communities [14]. The study was approved by the Towet community leaders, the Papua New Guinea National Research Institute, and the Western Sydney University Human Research Ethics Committee (H13179).

Risk management Dr. Hannah Sarvasy has been adopted as a clan member of the specific community, has been working with the community since 2011, and is fluent in the Nungon language. She has previously conducted language experiments in the community and has established very good relationships with the community. Her extensive knowledge of the community and cultural customs helped to ensure that local cultural values are respected in the design and conduct of the research. The other researchers received advance education by Dr. Sarvasy about local taboos to minimize the risk of participants being offended by the words or actions of a researcher.

Prior to travelling, all decisions regarding the research and the benefits and risks associated with the project were discussed and consulted by phone with local community leaders. No decisions were made without their approval and payment of local research assistants, participants and community members involved in any way with the research was in accordance with the local community leaders.

The experiment was designed to ensure minimum discomfort and a practice part was included in order for participants to familiarise themselves with the equipment. Participants could withdraw from the study at any time without affecting the relationship with the researcher. Participants were able to place the headphones on their heads themselves and adjust the volume accordingly, therefore ensuring the sound levels are comfortable to them. We ensured that, even at the maximum loudness setting, the acoustic levels were still moderate. Participants could stop the experiment and leave at any point without loss of remuneration.

Participation in the research was voluntary for community members and they were remunerated in local currency for their time, earning more than would be possible for a day's labor doing anything else in the region. However, their participation in the research does mean time away from their usual work activities doing farming, childcare, domestic activities, and house construction. Thus, care was taken to ensure that participants' time in the study was minimized as much as possible.

Prior to the fieldwork, management of personal safety risks involved comprehensive briefings by Dr. Hannah Sarvasy of all personnel to minimize the risk of researchers' inadvertently violating local codes of conduct (hence inciting anger). Precautions throughout the trip included continually checking with local community members and leaders to monitor any unanticipated safety risks and to ensure that the community was pleased with how the researchers were conducting themselves.

Inclusivity in global research

Ethical considerations, permits and authorship

This section is applicable to all research types.

Provide details as to who granted permissions and/or consent for the study to take place in the Methods section of your manuscript. This should include the names of **all** ethics boards, governmental organizations, community leaders or other bodies that provided approval for the study. If individuals provided approval refer to these people by their role or title but do not list their name(s).

Western Sydney University Ethics Human Research Ethics Committee
The National Research Institute (NRI) of Papua New Guinea
Towet community leaders

(also reported in the manuscript)

If there were any deviations from the study protocol after approval was obtained please provide details of these changes in the Methods section of your manuscript.

There were no deviations from the study protocol after approval was obtained.

Did this study involve local collaborators that are residents of the country where the research was conducted or members of the community studied? If you do not have any authors from said communities, please provide an explanation for this below.

The research was supported by local residents who hosted us and welcomed us into their communities. We received help with data collection from local research assistants, as described in the manuscript.

Everyone listed as an author should meet PLOS' criteria for authorship and all individuals who meet these criteria should be included in the author byline, rather than the acknowledgements. Authorship criteria is based on the International Committee of Medical Journal Editors (ICMJE) Uniform Requirements for Manuscripts Submitted to Biomedical Journals - for further information please see here: https://journals.plos.org/plosone/s/authorship.

Human subjects research (e.g. health research, medical research, cross-cultural psychology)

Did you obtain written informed consent from a representative of the local community or region before the research took place? How did you establish who speaks for the community? Details of written informed consent obtained from study participants should be reported separately in the Methods section of your manuscript.

Before the research took place, Dr. Hannah S. Sarvasy had regular contact by phone with the community leaders to discuss the research and the impact of the research on the community and received consent for the research over phone. Dr. Hannah Sarvasy has been adopted as a clan member of the specific community, has been working with the community since 2011, and is fluent in the Nungon language. She has previously conducted language experiments in the community and has established very good [illegible]

How did members of the local community provide input on the aims of the research investigation, its

Similar to above, Dr. Hannah S. Sarvasy had regular contact with the community leaders over phone before the research trip took place and discussed the aims of the research, the best suitable methods and anticipated outcomes with them.

methodology, and its anticipated outcome(s)?

When engaging with the local community, how did you ensure that the informed consent documents and other materials could be understood by local stakeholders?

Dr. Hannah S. Sarvasy is fluent in the local language, and helped to translate the necessary documents to the local research asisstants. The local research assistants also spoke some English, and were thus able to communicate with the researchers in English as well. The local research assistants translated the informed consent documents to participants.

Will the findings of the research be made available in an understandable format to stakeholders in the community where the study was conducted (e.g. via a presentation, summary report, copies of publications, etc.)? Please provide details of how this will be achieved.

We will provide copies of the publications to the community and aim to record a presentation on the research findings that can be shown in the community.

Non-human subjects research using specimens/ animals collected as part of the study, or those housed in archival collections. Examples include archaeology, paleontology, botany and zoology.

Did the permission you obtained from a local authority to perform the study include an agreement on access to outputs and benefit sharing? This may include procedures to enable fair distribution of the benefits and resources arising from the research performed. Please include any details of Prior Informed Consent and Benefit Sharing Agreements obtained. These may be required by field-specific regulations, for example the Convention on Biological Diversity (CBD) and the associated Nagoya Protocol.

NA

If the material used in your study was imported, please A) provide the year it was imported and B) indicate whether permits were obtained to import/export the materials used, C) provide details of any permits obtained. If this information is not available, please indicate this.

NA

If you used archival specimens, please state how the material used in your study was acquired by the institute it is held in and provide details of any permits obtained for the original excavations/ sample collection. If this information is not available, please indicate this.

NA

How was the potential cultural significance of the materials collected in your study to local communities considered in your research design? Were Indigenous peoples and/or local researchers and institutions involved with archaeological excavations / collection of specimens? If so, please provide a description of their involvement.

NA

If your manuscript includes photographs of human remains please indicate whether authors obtained permission from descendants or affiliated cultural communities to do so.

NA

I. Uruwa interview

The interview with participants from the Uruwa River Valley consisted of the following questions with possible answers provided in brackets:

1. What is your age?
2. Which village are you from?
3. Male/female?
4. Do you go to church? (Yes; no; sometimes)
5. Which do you go to? (SDA; Lutheran; I do not go to church)
6. When you go to church, do you sing songs or do you just listen? (I sing; I just listen)
7. Are you a song leader? (Yes; no)
8. Do you sing hymns outside of church? (Yes; no; sometimes)
9. When they sing songs, do you understand the words you sing?(Yes; no; sometimes)
10. Are you always happy or always sad when you hear all church songs, or are you happy when you hear some church songs and sad when you hear other songs? (Always happy; always sad; sometimes happy sometimes sad)
11. Why? Is the meaning of the words grabbing you that you feel sad or the sound? (Meaning of the words; the sound; meaning of the words and the sound)
12. Did you used to try different songs? (Yes; no)
13. What types of music did you used to play or sing? (*Biru* (a local flute); *uwing* (an hour glass shaped drum); guitar)
14. If a song seized you on the insides, how did it make you feel? (Happy; sad; sometimes happy sometimes sad)
15. When you used to sing a song in other peoples' language, would you understand? (Yes; no)
16. When you were small, how did the older people used to do songs or music? (*Biru*; *uwing*; guitar)
17. These days on your phone or radio, what songs do you find beautiful? (Open question, only answered if people have a phone or radio)
18. These days, do you listen to songs from other places or not? (Yes; no)
19. From where?

J. Full summaries of hypothesis-driven models

All models outputs show the following regression coefficients of the group-level and population-level effects:

- *Estimate*: mean coefficient of the posterior distribution.
- *Estimate error*: standard deviation of the posterior distribution.
- *l-95% CI* and *u-95% CI*: lower and upper 95% credibility intervals.
- *Rhat*: provides information on the convergence of the algorithm.
- *Bulk ESS* and *Tail ESS*: effective sample size measures.

Cadences

Uruwa: Minimal Exposure

```
 Family: bernoulli
  Links: mu = logit
Formula: bin_response ~ (cad_mode1 * cad_mode2 + diff_mean_pitch1_2) * timbre + (cad_mode1 * cad_mode2 + diff_mean_pitch1_2 | participant)
   Data: data_cut_min_cad (Number of observations: 204)
Samples: 4 chains, each with iter = 6000; warmup = 1000; thin = 1;
         total post-warmup samples = 20000

Group-Level Effects:
~participant (Number of levels: 17)
                                                   Estimate Est.Error l-95% CI u-95% CI Rhat Bulk_ESS Tail_ESS
sd(Intercept)                                          1.49      0.45     0.79     2.54 1.00     7129    11841
sd(cad_mode1Min)                                       0.37      0.26     0.01     0.99 1.00     7010     8490
sd(cad_mode2Min)                                       0.73      0.38     0.08     1.58 1.00     4897     5296
sd(diff_mean_pitch1_2)                                 0.38      0.26     0.02     1.00 1.00     7656     9302
sd(cad_mode1Min:cad_mode2Min)                          0.89      0.39     0.17     1.72 1.00     5619     4377
cor(Intercept,cad_mode1Min)                            0.17      0.39    -0.65     0.82 1.00    22749    14094
cor(Intercept,cad_mode2Min)                            0.10      0.35    -0.59     0.72 1.00    16658    14769
cor(cad_mode1Min,cad_mode2Min)                         0.16      0.40    -0.66     0.82 1.00     7750    11981
cor(Intercept,diff_mean_pitch1_2)                     -0.13      0.38    -0.79     0.63 1.00    21919    14806
cor(cad_mode1Min,diff_mean_pitch1_2)                  -0.15      0.41    -0.83     0.67 1.00    11844    14214
cor(cad_mode2Min,diff_mean_pitch1_2)                  -0.20      0.39    -0.83     0.61 1.00    15756    15359
cor(Intercept,cad_mode1Min:cad_mode2Min)              -0.11      0.33    -0.70     0.55 1.00    15792    13309
cor(cad_mode1Min,cad_mode1Min:cad_mode2Min)           -0.12      0.40    -0.80     0.67 1.00     6453    10324
cor(cad_mode2Min,cad_mode1Min:cad_mode2Min)           -0.17      0.36    -0.79     0.56 1.00    10349    13604
cor(diff_mean_pitch1_2,cad_mode1Min:cad_mode2Min)      0.13      0.39    -0.64     0.80 1.00     8607    13887

Population-Level Effects:
                                         Estimate Est.Error l-95% CI u-95% CI Rhat Bulk_ESS Tail_ESS
Intercept                                   -0.34      0.39    -1.12     0.43 1.00     8106    12061
cad_mode1Min                                -0.03      0.21    -0.44     0.39 1.00    21751    13844
cad_mode2Min                                 0.04      0.26    -0.48     0.55 1.00    14817    14192
diff_mean_pitch1_2                          -0.08      0.19    -0.47     0.30 1.00    18120    14937
timbreStrings                               -0.26      0.38    -1.05     0.47 1.00     8273    11123
cad_mode1Min:cad_mode2Min                   -0.08      0.28    -0.65     0.48 1.00    16278    14503
cad_mode1Min:timbreStrings                  -0.11      0.21    -0.53     0.31 1.00    20181    14765
cad_mode2Min:timbreStrings                   0.04      0.26    -0.48     0.58 1.00    15199    13781
diff_mean_pitch1_2:timbreStrings             0.11      0.19    -0.27     0.50 1.00    19703    14212
cad_mode1Min:cad_mode2Min:timbreStrings     -0.16      0.28    -0.74     0.40 1.00    15128    13988
```

Uruwa: SDA

```
 Family: bernoulli
  Links: mu = logit
Formula: bin_response ~ (cad_mode1 * cad_mode2 + diff_mean_pitch1_2) * timbre + (cad_mode1 * cad_mode2 + diff_mean_pitch1_2 | participant)
   Data: data_cut_SDA_cad (Number of observations: 708)
Samples: 4 chains, each with iter = 6000; warmup = 1000; thin = 1;
         total post-warmup samples = 20000

Group-Level Effects:
~participant (Number of levels: 59)
                                                   Estimate Est.Error l-95% CI u-95% CI Rhat Bulk_ESS Tail_ESS
sd(Intercept)                                          0.74      0.15     0.48     1.05 1.00     8100    13573
sd(cad_mode1Min)                                       0.36      0.15     0.04     0.66 1.00     5565     5432
sd(cad_mode2Min)                                       0.29      0.16     0.02     0.62 1.00     5062     8285
sd(diff_mean_pitch1_2)                                 0.22      0.14     0.01     0.51 1.00     5180     8528
sd(cad_mode1Min:cad_mode2Min)                          0.18      0.13     0.01     0.47 1.00     7194    10976
cor(Intercept,cad_mode1Min)                           -0.51      0.28    -0.91     0.20 1.00    13013    12363
cor(Intercept,cad_mode2Min)                            0.12      0.34    -0.58     0.73 1.00    20254    14025
cor(cad_mode1Min,cad_mode2Min)                        -0.08      0.37    -0.75     0.64 1.00    13181    14937
cor(Intercept,diff_mean_pitch1_2)                     -0.19      0.35    -0.79     0.57 1.00    19896    13956
cor(cad_mode1Min,diff_mean_pitch1_2)                  -0.03      0.38    -0.73     0.70 1.00    15828    15268
cor(cad_mode2Min,diff_mean_pitch1_2)                   0.04      0.39    -0.71     0.75 1.00    14152    15720
cor(Intercept,cad_mode1Min:cad_mode2Min)               0.11      0.38    -0.65     0.77 1.00    26118    14856
cor(cad_mode1Min,cad_mode1Min:cad_mode2Min)           -0.01      0.39    -0.74     0.73 1.00    20068    16054
cor(cad_mode2Min,cad_mode1Min:cad_mode2Min)            0.17      0.40    -0.66     0.83 1.00    14735    16272
cor(diff_mean_pitch1_2,cad_mode1Min:cad_mode2Min)      0.02      0.40    -0.74     0.76 1.00    17813    17206

Population-Level Effects:
                                         Estimate Est.Error l-95% CI u-95% CI Rhat Bulk_ESS Tail_ESS
Intercept                                    0.30      0.13     0.05     0.56 1.00    14574    15576
cad_mode1Min                                 0.19      0.10    -0.01     0.39 1.00    25081    14367
cad_mode2Min                                -0.34      0.10    -0.53    -0.15 1.00    28136    15841
diff_mean_pitch1_2                           0.08      0.08    -0.09     0.24 1.00    28267    15639
timbreStrings                                0.07      0.13    -0.18     0.32 1.00    13875    14882
cad_mode1Min:cad_mode2Min                    0.05      0.09    -0.12     0.23 1.00    36536    13526
cad_mode1Min:timbreStrings                  -0.15      0.10    -0.34     0.05 1.00    22530    15174
cad_mode2Min:timbreStrings                   0.03      0.10    -0.16     0.21 1.00    26998    15568
diff_mean_pitch1_2:timbreStrings             0.01      0.08    -0.15     0.17 1.00    31574    16344
cad_mode1Min:cad_mode2Min:timbreStrings     -0.07      0.09    -0.25     0.11 1.00    33116    15225
```

Uruwa: Lutheran

```
 Family: bernoulli
  Links: mu = logit
Formula: bin_response ~ (cad_mode1 * cad_mode2 + diff_mean_pitch1_2) * timbre + (cad_mode1 * cad_mode2 + diff_mean_pitch1_2 | participant)
   Data: data_cut_Lut_cad (Number of observations: 420)
Samples: 4 chains, each with iter = 6000; warmup = 1000; thin = 1;
         total post-warmup samples = 20000

Group-Level Effects:
```

```
~participant (Number of levels: 35)
                                                 Estimate Est.Error l-95% CI u-95% CI Rhat Bulk_ESS Tail_ESS
sd(Intercept)                                        1.07      0.26     0.62     1.64 1.00     6944    10791
sd(cad_mode1Min)                                     0.62      0.24     0.16     1.12 1.00     6023     5327
sd(cad_mode2Min)                                     1.01      0.26     0.57     1.57 1.00     8514    12922
sd(diff_mean_pitch1_2)                               0.64      0.22     0.22     1.10 1.00     6981     6938
sd(cad_mode1Min:cad_mode2Min)                        0.48      0.26     0.03     1.02 1.00     5001     7199
cor(Intercept,cad_mode1Min)                         -0.17      0.31    -0.72     0.46 1.00    14494    14483
cor(Intercept,cad_mode2Min)                          0.22      0.26    -0.31     0.68 1.00     9538    13266
cor(cad_mode1Min,cad_mode2Min)                      -0.22      0.31    -0.77     0.40 1.00     5369     9313
cor(Intercept,diff_mean_pitch1_2)                   -0.16      0.29    -0.68     0.42 1.00    13568    13820
cor(cad_mode1Min,diff_mean_pitch1_2)                 0.46      0.29    -0.20     0.89 1.00     7342     8780
cor(cad_mode2Min,diff_mean_pitch1_2)                -0.29      0.28    -0.78     0.30 1.00    14228    15383
cor(Intercept,cad_mode1Min:cad_mode2Min)             0.13      0.34    -0.57     0.74 1.00    17821    15368
cor(cad_mode1Min,cad_mode1Min:cad_mode2Min)          0.12      0.36    -0.61     0.77 1.00    11938    15330
cor(cad_mode2Min,cad_mode1Min:cad_mode2Min)         -0.18      0.34    -0.77     0.54 1.00    16161    16001
cor(diff_mean_pitch1_2,cad_mode1Min:cad_mode2Min)    0.05      0.35    -0.64     0.71 1.00    15338    16551

Population-Level Effects:
                                       Estimate Est.Error l-95% CI u-95% CI Rhat Bulk_ESS Tail_ESS
Intercept                                  0.24      0.22    -0.18     0.67 1.00    11705    12501
cad_mode1Min                               0.20      0.17    -0.13     0.54 1.00    17204    14438
cad_mode2Min                              -0.25      0.21    -0.66     0.17 1.00    14581    14940
diff_mean_pitch1_2                         0.27      0.16    -0.05     0.59 1.00    16322    14184
timbreStrings                              0.18      0.22    -0.25     0.62 1.00    13767    14072
cad_mode1Min:cad_mode2Min                  0.05      0.16    -0.25     0.36 1.00    18261    13069
cad_mode1Min:timbreStrings                -0.15      0.17    -0.49     0.18 1.00    17125    14386
cad_mode2Min:timbreStrings                 0.14      0.21    -0.27     0.57 1.00    15459    14019
diff_mean_pitch1_2:timbreStrings           0.21      0.16    -0.10     0.53 1.00    18068    14865
cad_mode1Min:cad_mode2Min:timbreStrings    0.06      0.15    -0.25     0.36 1.00    20420    14119
```

Sydney: Non-musician

```
 Family: bernoulli
  Links: mu = logit
Formula: bin_response ~ (cad_mode1 * cad_mode2 + diff_mean_pitch1_2) * timbre + (cad_mode1 * cad_mode2 + diff_mean_pitch1_2 | participant)
   Data: data_cut_nonmus_cad (Number of observations: 707)
Samples: 4 chains, each with iter = 6000; warmup = 1000; thin = 1;
         total post-warmup samples = 20000

Group-Level Effects:
~participant (Number of levels: 59)
                                                 Estimate Est.Error l-95% CI u-95% CI Rhat Bulk_ESS Tail_ESS
sd(Intercept)                                        0.67      0.19     0.31     1.05 1.00     6254     5529
sd(cad_mode1Min)                                     0.43      0.20     0.05     0.84 1.00     5372     6328
sd(cad_mode2Min)                                     0.83      0.19     0.48     1.23 1.00     7922    11725
sd(diff_mean_pitch1_2)                               0.74      0.18     0.42     1.12 1.00     8792    12294
sd(cad_mode1Min:cad_mode2Min)                        0.18      0.13     0.01     0.50 1.00     9692    11326
cor(Intercept,cad_mode1Min)                          0.16      0.33    -0.52     0.76 1.00    13262    12660
cor(Intercept,cad_mode2Min)                         -0.07      0.27    -0.59     0.46 1.00     7144    10142
cor(cad_mode1Min,cad_mode2Min)                      -0.34      0.31    -0.84     0.36 1.00     4492     5743
cor(Intercept,diff_mean_pitch1_2)                    0.34      0.25    -0.19     0.78 1.00     7041    10791
cor(cad_mode1Min,diff_mean_pitch1_2)                 0.36      0.31    -0.33     0.85 1.00     4582     6721
cor(cad_mode2Min,diff_mean_pitch1_2)                -0.39      0.23    -0.79     0.09 1.00     9530    13450
cor(Intercept,cad_mode1Min:cad_mode2Min)             0.10      0.39    -0.67     0.79 1.00    29254    16449
cor(cad_mode1Min,cad_mode1Min:cad_mode2Min)          0.03      0.41    -0.74     0.77 1.00    24195    15481
cor(cad_mode2Min,cad_mode1Min:cad_mode2Min)         -0.05      0.39    -0.76     0.71 1.00    28930    17015
cor(diff_mean_pitch1_2,cad_mode1Min:cad_mode2Min)    0.09      0.39    -0.68     0.78 1.00    26206    17501

Population-Level Effects:
                                       Estimate Est.Error l-95% CI u-95% CI Rhat Bulk_ESS Tail_ESS
Intercept                                 -0.19      0.14    -0.49     0.08 1.00    18176    15136
cad_mode1Min                               0.57      0.14     0.31     0.85 1.00    14951    14771
cad_mode2Min                              -1.17      0.17    -1.51    -0.86 1.00    15093    13774
diff_mean_pitch1_2                         0.91      0.15     0.63     1.22 1.00    16336    14524
timbreStrings                             -0.11      0.14    -0.38     0.16 1.00    22369    16575
cad_mode1Min:cad_mode2Min                 -0.04      0.12    -0.26     0.19 1.00    30954    15905
cad_mode1Min:timbreStrings                -0.09      0.13    -0.34     0.16 1.00    27487    16023
cad_mode2Min:timbreStrings                -0.29      0.16    -0.60     0.02 1.00    21137    15313
diff_mean_pitch1_2:timbreStrings          -0.13      0.14    -0.42     0.14 1.00    19687    15516
cad_mode1Min:cad_mode2Min:timbreStrings    0.05      0.11    -0.17     0.26 1.00    38298    15090
```

Sydney: Musician

```
 Family: bernoulli
  Links: mu = logit
Formula: bin_response ~ (cad_mode1 * cad_mode2 + diff_mean_pitch1_2) * timbre + (cad_mode1 * cad_mode2 + diff_mean_pitch1_2 | participant)
   Data: data_cut_mus_cad (Number of observations: 228)
Samples: 4 chains, each with iter = 6000; warmup = 1000; thin = 1;
         total post-warmup samples = 20000

Group-Level Effects:
~participant (Number of levels: 19)
                                                 Estimate Est.Error l-95% CI u-95% CI Rhat Bulk_ESS Tail_ESS
sd(Intercept)                                        0.92      0.54     0.07     2.11 1.00     5469     7257
sd(cad_mode1Min)                                     0.49      0.39     0.02     1.44 1.00    11137    10555
sd(cad_mode2Min)                                     1.50      0.62     0.42     2.90 1.00     5736     5340
sd(diff_mean_pitch1_2)                               0.56      0.39     0.03     1.47 1.00     7421     9634
sd(cad_mode1Min:cad_mode2Min)                        0.87      0.53     0.05     2.05 1.00     5195     8069
cor(Intercept,cad_mode1Min)                         -0.02      0.40    -0.76     0.74 1.00    26271    14540
cor(Intercept,cad_mode2Min)                          0.06      0.36    -0.66     0.72 1.00     7864    10894
cor(cad_mode1Min,cad_mode2Min)                      -0.11      0.41    -0.81     0.69 1.00     5776    10557
cor(Intercept,diff_mean_pitch1_2)                    0.11      0.39    -0.66     0.79 1.00    16706    15032
cor(cad_mode1Min,diff_mean_pitch1_2)                -0.00      0.41    -0.75     0.75 1.00    12355    14873
cor(cad_mode2Min,diff_mean_pitch1_2)                 0.15      0.38    -0.63     0.80 1.00    19486    16275
cor(Intercept,cad_mode1Min:cad_mode2Min)            -0.15      0.39    -0.80     0.65 1.00    12968    13736
cor(cad_mode1Min,cad_mode1Min:cad_mode2Min)          0.00      0.40    -0.74     0.74 1.00    12897    14352
cor(cad_mode2Min,cad_mode1Min:cad_mode2Min)          0.07      0.37    -0.65     0.74 1.00    18117    16826
cor(diff_mean_pitch1_2,cad_mode1Min:cad_mode2Min)   -0.14      0.39    -0.81     0.66 1.00    12673    15804

Population-Level Effects:
                                       Estimate Est.Error l-95% CI u-95% CI Rhat Bulk_ESS Tail_ESS
Intercept                                  0.15      0.41    -0.65     0.97 1.00    15437    13678
cad_mode1Min                               1.74      0.49     0.93     2.86 1.00    10619     8887
cad_mode2Min                              -2.74      0.65    -4.20    -1.63 1.00     9584     9720
diff_mean_pitch1_2                         1.15      0.30     0.61     1.78 1.00    18811    12885
timbreStrings                             -0.26      0.39    -1.05     0.49 1.00    16315    13681
cad_mode1Min:cad_mode2Min                 -0.48      0.41    -1.31     0.28 1.00    15478    12879
cad_mode1Min:timbreStrings                -0.35      0.37    -1.11     0.35 1.00    15757    12856
cad_mode2Min:timbreStrings                -0.12      0.47    -1.05     0.82 1.00    15050    13138
diff_mean_pitch1_2:timbreStrings          -0.13      0.27    -0.68     0.40 1.00    22119    14451
cad_mode1Min:cad_mode2Min:timbreStrings    0.27      0.39    -0.50     1.04 1.00    16592    13544
```

Melodies

Uruwa: Minimal Exposure

```
 Family: bernoulli
  Links: mu = logit
Formula: bin_response ~ diff_mean_pitch1_2 * timbre * melody + (diff_mean_pitch1_2 * melody | participant)
   Data: data_cut_min_mel (Number of observations: 569)
Samples: 4 chains, each with iter = 6000; warmup = 1000; thin = 1;
         total post-warmup samples = 20000

Group-Level Effects:
~participant (Number of levels: 19)
                                                                 Estimate Est.Error l-95% CI u-95% CI Rhat Bulk_ESS Tail_ESS
sd(Intercept)                                                        1.67      0.36     1.09     2.50 1.00     8811    13064
sd(diff_mean_pitch1_2)                                               0.79      0.51     0.04     1.94 1.00     7225    10484
sd(melody1)                                                          0.37      0.22     0.02     0.86 1.00     7735    10293
sd(melody2)                                                          0.19      0.15     0.01     0.56 1.00    12594    12283
sd(diff_mean_pitch1_2:melody1)                                       0.74      0.55     0.03     2.04 1.00    10327    12235
sd(diff_mean_pitch1_2:melody2)                                       0.67      0.53     0.02     1.98 1.00    11540    10538
cor(Intercept,diff_mean_pitch1_2)                                   -0.02      0.35    -0.68     0.64 1.00    32339    13839
cor(Intercept,melody1)                                               0.02      0.35    -0.65     0.66 1.00    33150    15275
cor(diff_mean_pitch1_2,melody1)                                     -0.20      0.37    -0.81     0.57 1.00    13825    15086
cor(Intercept,melody2)                                              -0.08      0.38    -0.76     0.66 1.00    39201    14848
cor(diff_mean_pitch1_2,melody2)                                     -0.04      0.38    -0.73     0.68 1.00    22316    16353
cor(melody1,melody2)                                                -0.05      0.38    -0.73     0.68 1.00    22604    17223
cor(Intercept,diff_mean_pitch1_2:melody1)                           -0.17      0.37    -0.78     0.60 1.00    30002    15765
cor(diff_mean_pitch1_2,diff_mean_pitch1_2:melody1)                  -0.04      0.38    -0.73     0.68 1.00    21651    15847
cor(melody1,diff_mean_pitch1_2:melody1)                              0.07      0.37    -0.66     0.74 1.00    19536    15958
cor(melody2,diff_mean_pitch1_2:melody1)                              0.02      0.38    -0.69     0.72 1.00    15356    17038
cor(Intercept,diff_mean_pitch1_2:melody2)                           -0.03      0.37    -0.71     0.68 1.00    39020    13507
cor(diff_mean_pitch1_2,diff_mean_pitch1_2:melody2)                   0.01      0.37    -0.70     0.70 1.00    25178    16121
cor(melody1,diff_mean_pitch1_2:melody2)                             -0.03      0.38    -0.72     0.69 1.00    22007    16648
cor(melody2,diff_mean_pitch1_2:melody2)                              0.02      0.38    -0.70     0.73 1.00    15502    16308
cor(diff_mean_pitch1_2:melody1,diff_mean_pitch1_2:melody2)          -0.09      0.38    -0.77     0.65 1.00    14994    17365

Population-Level Effects:
                                         Estimate Est.Error l-95% CI u-95% CI Rhat Bulk_ESS Tail_ESS
Intercept                                   -0.27      0.38    -1.01     0.47 1.00     6732     9703
diff_mean_pitch1_2                           0.09      0.37    -0.65     0.80 1.00    27148    16002
timbreStrings                               -0.09      0.37    -0.83     0.63 1.00     6765    10553
melody1                                     -0.27      0.19    -0.64     0.10 1.00    23084    16458
melody2                                      0.12      0.17    -0.21     0.44 1.00    25292    16387
diff_mean_pitch1_2:timbreStrings            -0.13      0.37    -0.87     0.61 1.00    25233    14017
diff_mean_pitch1_2:melody1                  -0.20      0.46    -1.09     0.69 1.00    27389    16492
diff_mean_pitch1_2:melody2                  -0.30      0.50    -1.30     0.68 1.00    29897    16196
timbreStrings:melody1                       -0.09      0.19    -0.46     0.28 1.00    22431    15332
timbreStrings:melody2                        0.03      0.17    -0.29     0.36 1.00    25365    16099
diff_mean_pitch1_2:timbreStrings:melody1    -0.50      0.47    -1.42     0.43 1.00    27184    15644
diff_mean_pitch1_2:timbreStrings:melody2     0.52      0.50    -0.44     1.53 1.00    29156    16242
```

Uruwa: SDA

```
 Family: bernoulli
  Links: mu = logit
Formula: bin_response ~ diff_mean_pitch1_2 * timbre * melody + (diff_mean_pitch1_2 * melody | participant)
   Data: data_cut_SDA_mel (Number of observations: 2009)
Samples: 4 chains, each with iter = 6000; warmup = 1000; thin = 1;
         total post-warmup samples = 20000

Group-Level Effects:
~participant (Number of levels: 67)
                                                                 Estimate Est.Error l-95% CI u-95% CI Rhat Bulk_ESS Tail_ESS
sd(Intercept)                                                        1.20      0.14     0.95     1.50 1.00     6012    10377
sd(diff_mean_pitch1_2)                                               0.52      0.29     0.03     1.11 1.00     4403     6569
sd(melody1)                                                          0.21      0.13     0.01     0.48 1.00     4559     8054
sd(melody2)                                                          0.36      0.14     0.06     0.62 1.00     3540     3742
sd(diff_mean_pitch1_2:melody1)                                       0.36      0.26     0.02     0.97 1.00     8049     9868
sd(diff_mean_pitch1_2:melody2)                                       0.70      0.42     0.04     1.56 1.00     4882     8044
cor(Intercept,diff_mean_pitch1_2)                                    0.04      0.31    -0.57     0.62 1.00    24169    13599
cor(Intercept,melody1)                                              -0.07      0.33    -0.68     0.60 1.00    24743    13400
cor(diff_mean_pitch1_2,melody1)                                      0.03      0.36    -0.67     0.71 1.00    11575    13787
cor(Intercept,melody2)                                              -0.01      0.27    -0.52     0.51 1.00    20636    14545
cor(diff_mean_pitch1_2,melody2)                                     -0.28      0.34    -0.82     0.50 1.00     4326     7337
cor(melody1,melody2)                                                -0.31      0.37    -0.85     0.53 1.00     5145     9286
cor(Intercept,diff_mean_pitch1_2:melody1)                            0.11      0.37    -0.63     0.76 1.00    28149    15060
cor(diff_mean_pitch1_2,diff_mean_pitch1_2:melody1)                   0.07      0.37    -0.66     0.74 1.00    17326    14954
cor(melody1,diff_mean_pitch1_2:melody1)                              0.10      0.38    -0.65     0.77 1.00    15905    15754
cor(melody2,diff_mean_pitch1_2:melody1)                             -0.13      0.37    -0.77     0.61 1.00    17644    17557
cor(Intercept,diff_mean_pitch1_2:melody2)                           -0.09      0.32    -0.67     0.55 1.00    25475    14602
cor(diff_mean_pitch1_2,diff_mean_pitch1_2:melody2)                  -0.12      0.36    -0.75     0.60 1.00    10018    13266
cor(melody1,diff_mean_pitch1_2:melody2)                              0.08      0.37    -0.65     0.74 1.00    10153    13600
cor(melody2,diff_mean_pitch1_2:melody2)                              0.15      0.35    -0.59     0.76 1.00    12336    14695
cor(diff_mean_pitch1_2:melody1,diff_mean_pitch1_2:melody2)          -0.08      0.38    -0.75     0.66 1.00    11633    15987

Population-Level Effects:
                                         Estimate Est.Error l-95% CI u-95% CI Rhat Bulk_ESS Tail_ESS
Intercept                                    0.24      0.15    -0.06     0.54 1.00     4293     7743
diff_mean_pitch1_2                           0.22      0.18    -0.13     0.57 1.00    24789    15624
timbreStrings                               -0.10      0.16    -0.40     0.21 1.00     4006     7307
melody1                                      0.02      0.08    -0.14     0.17 1.00    21601    15094
melody2                                     -0.19      0.09    -0.36    -0.01 1.00    19692    15467
diff_mean_pitch1_2:timbreStrings             0.07      0.18    -0.28     0.42 1.00    25564    15477
diff_mean_pitch1_2:melody1                  -0.09      0.24    -0.55     0.37 1.00    20337    16116
diff_mean_pitch1_2:melody2                   0.00      0.27    -0.53     0.54 1.00    21054    16008
timbreStrings:melody1                       -0.11      0.08    -0.27     0.05 1.00    22395    16628
timbreStrings:melody2                       -0.14      0.09    -0.32     0.03 1.00    19617    15558
diff_mean_pitch1_2:timbreStrings:melody1     0.11      0.23    -0.35     0.56 1.00    20646    16899
diff_mean_pitch1_2:timbreStrings:melody2    -0.32      0.27    -0.85     0.22 1.00    19932    15770
```

Uruwa: Lutheran

```
 Family: bernoulli
  Links: mu = logit
Formula: bin_response ~ diff_mean_pitch1_2 * timbre * melody + (diff_mean_pitch1_2 * melody | participant)
   Data: data_cut_Lut_mel (Number of observations: 1080)
Samples: 4 chains, each with iter = 6000; warmup = 1000; thin = 1;
         total post-warmup samples = 20000

Group-Level Effects:
~participant (Number of levels: 36)
                                                                 Estimate Est.Error l-95% CI u-95% CI Rhat Bulk_ESS Tail_ESS
sd(Intercept)                                                        0.81      0.15     0.56     1.13 1.00     7982    12860
sd(diff_mean_pitch1_2)                                               0.49      0.33     0.02     1.22 1.00     6619    10245
sd(melody1)                                                          0.22      0.14     0.01     0.53 1.00     6798     9587
sd(melody2)                                                          0.29      0.16     0.02     0.62 1.00     5913     8387
```

```
sd(diff_mean_pitch1_2:melody1)                                   0.52      0.37     0.02     1.38 1.00     7408    10666
sd(diff_mean_pitch1_2:melody2)                                   0.50      0.37     0.02     1.36 1.00     9283     9796
cor(Intercept,diff_mean_pitch1_2)                               -0.09      0.34    -0.71     0.61 1.00    28227    14979
cor(Intercept,melody1)                                           0.01      0.34    -0.64     0.66 1.00    24659    13637
cor(diff_mean_pitch1_2,melody1)                                 -0.03      0.37    -0.72     0.68 1.00    14226    15370
cor(Intercept,melody2)                                           0.06      0.32    -0.57     0.65 1.00    24593    14887
cor(diff_mean_pitch1_2,melody2)                                  0.05      0.37    -0.66     0.72 1.00    12166    14730
cor(melody1,melody2)                                            -0.04      0.37    -0.72     0.68 1.00    12493    14971
cor(Intercept,diff_mean_pitch1_2:melody1)                        0.05      0.35    -0.63     0.70 1.00    28845    15031
cor(diff_mean_pitch1_2,diff_mean_pitch1_2:melody1)              -0.07      0.38    -0.74     0.67 1.00    18179    15459
cor(melody1,diff_mean_pitch1_2:melody1)                         -0.01      0.37    -0.70     0.69 1.00    17556    16739
cor(melody2,diff_mean_pitch1_2:melody1)                         -0.00      0.37    -0.70     0.70 1.00    17872    16387
cor(Intercept,diff_mean_pitch1_2:melody2)                        0.08      0.37    -0.65     0.74 1.00    32708    15606
cor(diff_mean_pitch1_2,diff_mean_pitch1_2:melody2)              -0.00      0.38    -0.71     0.71 1.00    19467    16079
cor(melody1,diff_mean_pitch1_2:melody2)                          0.08      0.38    -0.66     0.76 1.00    18991    16815
cor(melody2,diff_mean_pitch1_2:melody2)                          0.08      0.37    -0.66     0.75 1.00    16895    16863
cor(diff_mean_pitch1_2:melody1,diff_mean_pitch1_2:melody2)      -0.09      0.38    -0.76     0.65 1.00    12048    16786

Population-Level Effects:
                                         Estimate Est.Error l-95% CI u-95% CI Rhat Bulk_ESS Tail_ESS
Intercept                                    0.25      0.15    -0.05     0.54 1.00     8699    11706
diff_mean_pitch1_2                           0.51      0.23     0.06     0.97 1.00    24208    15333
timbreStrings                                0.13      0.15    -0.17     0.42 1.00     8380    11781
melody1                                     -0.26      0.11    -0.48    -0.06 1.00    26314    15988
melody2                                      0.02      0.11    -0.20     0.24 1.00    23842    15780
diff_mean_pitch1_2:timbreStrings             0.02      0.23    -0.43     0.48 1.00    25956    14827
diff_mean_pitch1_2:melody1                  -0.54      0.31    -1.14     0.06 1.00    23098    16038
diff_mean_pitch1_2:melody2                   0.16      0.33    -0.48     0.82 1.00    25423    16501
timbreStrings:melody1                       -0.06      0.11    -0.27     0.15 1.00    25285    15635
timbreStrings:melody2                        0.20      0.11    -0.02     0.42 1.00    22430    15401
diff_mean_pitch1_2:timbreStrings:melody1    -0.12      0.30    -0.72     0.46 1.00    24029    16096
diff_mean_pitch1_2:timbreStrings:melody2    -0.18      0.33    -0.83     0.49 1.00    26203    16956
```

Sydney: Non-musician

```
 Family: bernoulli
  Links: mu = logit
Formula: bin_response ~ diff_mean_pitch1_2 * timbre * melody + (diff_mean_pitch1_2 * melody | participant)
   Data: data_cut_nonmus_mel (Number of observations: 1800)
Samples: 4 chains, each with iter = 6000; warmup = 1000; thin = 1;
         total post-warmup samples = 20000

Group-Level Effects:
~participant (Number of levels: 60)
                                                              Estimate Est.Error l-95% CI u-95% CI Rhat Bulk_ESS Tail_ESS
sd(Intercept)                                                    0.46      0.09     0.29     0.64 1.00     8977    11732
sd(diff_mean_pitch1_2)                                           1.65      0.29     1.12     2.26 1.00     9595    13038
sd(melody1)                                                      0.18      0.12     0.01     0.44 1.00     5970     8673
sd(melody2)                                                      0.25      0.13     0.02     0.51 1.00     4570     8272
sd(diff_mean_pitch1_2:melody1)                                   0.44      0.32     0.02     1.17 1.00     6448    10234
sd(diff_mean_pitch1_2:melody2)                                   0.52      0.35     0.02     1.31 1.00     7901    10363
cor(Intercept,diff_mean_pitch1_2)                                0.17      0.21    -0.25     0.57 1.00     5856    10303
cor(Intercept,melody1)                                          -0.10      0.34    -0.72     0.58 1.00    21051    14784
cor(diff_mean_pitch1_2,melody1)                                 -0.22      0.34    -0.78     0.53 1.00    18700    15612
cor(Intercept,melody2)                                           0.19      0.32    -0.50     0.74 1.00    16551    14388
cor(diff_mean_pitch1_2,melody2)                                 -0.06      0.31    -0.64     0.56 1.00    18373    13705
cor(melody1,melody2)                                            -0.00      0.36    -0.68     0.69 1.00    10329    12628
cor(Intercept,diff_mean_pitch1_2:melody1)                        0.11      0.35    -0.60     0.73 1.00    23496    14586
cor(diff_mean_pitch1_2,diff_mean_pitch1_2:melody1)              -0.07      0.35    -0.71     0.63 1.00    23130    16080
cor(melody1,diff_mean_pitch1_2:melody1)                          0.01      0.37    -0.70     0.70 1.00    18074    16103
cor(melody2,diff_mean_pitch1_2:melody1)                          0.01      0.37    -0.69     0.70 1.00    17710    15792
cor(Intercept,diff_mean_pitch1_2:melody2)                       -0.12      0.35    -0.73     0.59 1.00    22909    14890
cor(diff_mean_pitch1_2,diff_mean_pitch1_2:melody2)               0.26      0.35    -0.51     0.82 1.00    19079    14422
cor(melody1,diff_mean_pitch1_2:melody2)                         -0.08      0.37    -0.74     0.65 1.00    17159    16251
cor(melody2,diff_mean_pitch1_2:melody2)                          0.04      0.36    -0.66     0.71 1.00    17447    16557
cor(diff_mean_pitch1_2:melody1,diff_mean_pitch1_2:melody2)      -0.11      0.38    -0.77     0.64 1.00    14939    17161

Population-Level Effects:
                                         Estimate Est.Error l-95% CI u-95% CI Rhat Bulk_ESS Tail_ESS
Intercept                                   -0.23      0.08    -0.39    -0.08 1.00    14598    15469
diff_mean_pitch1_2                           2.06      0.28     1.51     2.61 1.00    13490    14078
timbreStrings                               -0.08      0.08    -0.24     0.08 1.00    14099    15091
melody1                                     -0.34      0.09    -0.51    -0.17 1.00    22460    16708
melody2                                      0.02      0.09    -0.15     0.19 1.00    23138    16458
diff_mean_pitch1_2:timbreStrings            -0.09      0.27    -0.62     0.43 1.00    14715    14848
diff_mean_pitch1_2:melody1                  -0.12      0.25    -0.60     0.37 1.00    24032    16693
diff_mean_pitch1_2:melody2                  -0.21      0.27    -0.74     0.33 1.00    23833    15288
timbreStrings:melody1                        0.09      0.09    -0.08     0.26 1.00    23357    15873
timbreStrings:melody2                       -0.05      0.09    -0.22     0.12 1.00    23000    16195
diff_mean_pitch1_2:timbreStrings:melody1    -0.15      0.24    -0.62     0.32 1.00    23844    17008
diff_mean_pitch1_2:timbreStrings:melody2     0.27      0.27    -0.27     0.81 1.00    23715    16325
```

Sydney: Musician

```
 Family: bernoulli
  Links: mu = logit
Formula: bin_response ~ diff_mean_pitch1_2 * timbre * melody + (diff_mean_pitch1_2 * melody | participant)
   Data: data_cut_mus_mel (Number of observations: 570)
Samples: 4 chains, each with iter = 6000; warmup = 1000; thin = 1;
         total post-warmup samples = 20000

Group-Level Effects:
~participant (Number of levels: 19)
                                                              Estimate Est.Error l-95% CI u-95% CI Rhat Bulk_ESS Tail_ESS
sd(Intercept)                                                    0.23      0.16     0.01     0.61 1.00     8600    10549
sd(diff_mean_pitch1_2)                                           2.94      0.82     1.59     4.79 1.00    10695    14273
sd(melody1)                                                      0.21      0.16     0.01     0.60 1.00    13539    11518
sd(melody2)                                                      0.29      0.21     0.01     0.78 1.00     8710    10115
sd(diff_mean_pitch1_2:melody1)                                   0.84      0.66     0.03     2.46 1.00    10510    10277
sd(diff_mean_pitch1_2:melody2)                                   0.96      0.74     0.04     2.75 1.00    10280    11004
cor(Intercept,diff_mean_pitch1_2)                               -0.12      0.35    -0.75     0.59 1.00     5262     9588
cor(Intercept,melody1)                                           0.05      0.38    -0.67     0.74 1.00    27396    15398
cor(diff_mean_pitch1_2,melody1)                                 -0.11      0.37    -0.76     0.62 1.00    26576    15391
cor(Intercept,melody2)                                           0.09      0.38    -0.66     0.75 1.00    20834    15220
cor(diff_mean_pitch1_2,melody2)                                 -0.03      0.35    -0.69     0.65 1.00    25071    15867
cor(melody1,melody2)                                            -0.06      0.39    -0.75     0.69 1.00    15977    15248
cor(Intercept,diff_mean_pitch1_2:melody1)                        0.02      0.38    -0.70     0.72 1.00    23941    14843
cor(diff_mean_pitch1_2,diff_mean_pitch1_2:melody1)              -0.08      0.36    -0.73     0.63 1.00    25761    14801
cor(melody1,diff_mean_pitch1_2:melody1)                          0.01      0.38    -0.71     0.71 1.00    17802    14954
cor(melody2,diff_mean_pitch1_2:melody1)                          0.03      0.37    -0.69     0.72 1.00    17536    16425
cor(Intercept,diff_mean_pitch1_2:melody2)                        0.00      0.38    -0.69     0.70 1.00    24590    15259
cor(diff_mean_pitch1_2,diff_mean_pitch1_2:melody2)               0.05      0.36    -0.65     0.72 1.00    27809    16041
cor(melody1,diff_mean_pitch1_2:melody2)                         -0.05      0.38    -0.74     0.67 1.00    18886    16738
cor(melody2,diff_mean_pitch1_2:melody2)                          0.00      0.37    -0.69     0.70 1.00    17361    16714
cor(diff_mean_pitch1_2:melody1,diff_mean_pitch1_2:melody2)      -0.04      0.38    -0.73     0.70 1.00    15226    17486

Population-Level Effects:
```

```
                                          Estimate Est.Error l-95% CI u-95% CI Rhat Bulk_ESS Tail_ESS
Intercept                                    -0.23      0.14    -0.52     0.04 1.00    22856    14799
diff_mean_pitch1_2                            6.31      0.94     4.49     8.20 1.00    12806    13675
timbreStrings                                -0.04      0.14    -0.31     0.24 1.00    22827    16207
melody1                                      -0.41      0.20    -0.80    -0.03 1.00    23866    15756
melody2                                       0.33      0.20    -0.05     0.72 1.00    21919    14392
diff_mean_pitch1_2:timbreStrings              0.33      0.67    -0.97     1.71 1.00    18556    14751
diff_mean_pitch1_2:melody1                   -0.64      0.60    -1.85     0.51 1.00    24799    16350
diff_mean_pitch1_2:melody2                    0.03      0.61    -1.15     1.28 1.00    24922    14663
timbreStrings:melody1                         0.28      0.19    -0.09     0.66 1.00    21906    15283
timbreStrings:melody2                         0.08      0.19    -0.29     0.46 1.00    21736    16405
diff_mean_pitch1_2:timbreStrings:melody1     -0.42      0.59    -1.61     0.70 1.00    25977    15640
diff_mean_pitch1_2:timbreStrings:melody2      0.15      0.59    -1.01     1.33 1.00    26532    15948
```

Cadences and melodies

Uruwa: Minimal

```
 Family: bernoulli
  Links: mu = logit
Formula: bin_response ~ diff_mean_pitch1_2 * timbre + (diff_mean_pitch1_2 | participant)
   Data: data_cut_min_cad_mel (Number of observations: 773)
Samples: 4 chains, each with iter = 6000; warmup = 1000; thin = 1;
         total post-warmup samples = 20000

Group-Level Effects:
~participant (Number of levels: 19)
                                  Estimate Est.Error l-95% CI u-95% CI Rhat Bulk_ESS Tail_ESS
sd(Intercept)                         1.34      0.28     0.89     1.98 1.00     4922     9299
sd(diff_mean_pitch1_2)                0.21      0.16     0.01     0.59 1.00     7930     7598
cor(Intercept,diff_mean_pitch1_2)    -0.19      0.54    -0.97     0.89 1.00    15485    11230

Population-Level Effects:
                                 Estimate Est.Error l-95% CI u-95% CI Rhat Bulk_ESS Tail_ESS
Intercept                           -0.25      0.31    -0.87     0.35 1.00     3427     6853
diff_mean_pitch1_2                  -0.05      0.14    -0.33     0.23 1.00    17970    13600
timbreStrings                       -0.11      0.31    -0.73     0.50 1.00     3960     7098
diff_mean_pitch1_2:timbreStrings     0.04      0.14    -0.23     0.32 1.00    18456    13748
```

Uruwa: SDA

```
 Family: bernoulli
  Links: mu = logit
Formula: bin_response ~ diff_mean_pitch1_2 * timbre + (diff_mean_pitch1_2 | participant)
   Data: data_cut_SDA_cad_mel (Number of observations: 2717)
Samples: 4 chains, each with iter = 6000; warmup = 1000; thin = 1;
         total post-warmup samples = 20000

Group-Level Effects:
~participant (Number of levels: 69)
                                  Estimate Est.Error l-95% CI u-95% CI Rhat Bulk_ESS Tail_ESS
sd(Intercept)                         1.04      0.12     0.82     1.30 1.00     4468     7632
sd(diff_mean_pitch1_2)                0.13      0.10     0.01     0.36 1.00     5351     6895
cor(Intercept,diff_mean_pitch1_2)     0.04      0.52    -0.91     0.92 1.00    17215    12110

Population-Level Effects:
                                 Estimate Est.Error l-95% CI u-95% CI Rhat Bulk_ESS Tail_ESS
Intercept                            0.24      0.13    -0.02     0.50 1.00     2647     5101
diff_mean_pitch1_2                   0.14      0.07     0.01     0.27 1.00    19705    14658
timbreStrings                       -0.01      0.13    -0.26     0.26 1.00     2456     5022
diff_mean_pitch1_2:timbreStrings     0.01      0.07    -0.12     0.14 1.00    22652    14540
```

Uruwa: Lutheran

```
 Family: bernoulli
  Links: mu = logit
Formula: bin_response ~ diff_mean_pitch1_2 * timbre + (diff_mean_pitch1_2 | participant)
   Data: data_cut_Lut_cad_mel (Number of observations: 1500)
Samples: 4 chains, each with iter = 6000; warmup = 1000; thin = 1;
         total post-warmup samples = 20000

Group-Level Effects:
~participant (Number of levels: 38)
                                  Estimate Est.Error l-95% CI u-95% CI Rhat Bulk_ESS Tail_ESS
sd(Intercept)                         0.77      0.13     0.54     1.05 1.00     5797    10015
sd(diff_mean_pitch1_2)                0.38      0.16     0.05     0.71 1.00     4077     4731
cor(Intercept,diff_mean_pitch1_2)    -0.22      0.36    -0.86     0.53 1.00    10202     7566

Population-Level Effects:
                                 Estimate Est.Error l-95% CI u-95% CI Rhat Bulk_ESS Tail_ESS
Intercept                            0.23      0.14    -0.04     0.50 1.00     4575     7672
diff_mean_pitch1_2                   0.27      0.11     0.05     0.49 1.00    14036    13570
timbreStrings                        0.16      0.14    -0.10     0.44 1.00     4551     7589
diff_mean_pitch1_2:timbreStrings     0.11      0.11    -0.11     0.32 1.00    13592    13205
```

Sydney: Non-musician

```
 Family: bernoulli
  Links: mu = logit
Formula: bin_response ~ diff_mean_pitch1_2 * timbre + (diff_mean_pitch1_2 | participant)
   Data: data_cut_nonmus_cad_mel (Number of observations: 2507)
Samples: 4 chains, each with iter = 6000; warmup = 1000; thin = 1;
         total post-warmup samples = 20000

Group-Level Effects:
~participant (Number of levels: 60)
                                  Estimate Est.Error l-95% CI u-95% CI Rhat Bulk_ESS Tail_ESS
sd(Intercept)                         0.33      0.07     0.21     0.47 1.00     9127    12083
sd(diff_mean_pitch1_2)                0.67      0.12     0.45     0.92 1.00     8994    12190
cor(Intercept,diff_mean_pitch1_2)     0.67      0.19     0.24     0.97 1.00     3589     5142

Population-Level Effects:
                                 Estimate Est.Error l-95% CI u-95% CI Rhat Bulk_ESS Tail_ESS
Intercept                           -0.19      0.06    -0.31    -0.07 1.00    11123    13796
diff_mean_pitch1_2                   1.02      0.12     0.80     1.26 1.00    11613    13458
timbreStrings                       -0.07      0.06    -0.19     0.05 1.00    11969    14330
diff_mean_pitch1_2:timbreStrings    -0.12      0.11    -0.35     0.10 1.00    11343    13630
```

Sydney: Musician

```
 Family: bernoulli
  Links: mu = logit
Formula: bin_response ~ diff_mean_pitch1_2 * timbre + (diff_mean_pitch1_2 | participant)
   Data: data_cut_mus_cad_mel (Number of observations: 798)
Samples: 4 chains, each with iter = 6000; warmup = 1000; thin = 1;
         total post-warmup samples = 20000

Group-Level Effects:
~participant (Number of levels: 19)
                                  Estimate Est.Error l-95% CI u-95% CI Rhat Bulk_ESS Tail_ESS
sd(Intercept)                         0.10      0.08     0.00     0.30 1.00    10343     9553
sd(diff_mean_pitch1_2)                0.35      0.23     0.02     0.87 1.00     6167     7725
cor(Intercept,diff_mean_pitch1_2)     0.03      0.57    -0.94     0.95 1.00     7252    11086

Population-Level Effects:
                                 Estimate Est.Error l-95% CI u-95% CI Rhat Bulk_ESS Tail_ESS
Intercept                           -0.10      0.09    -0.27     0.07 1.00    20481    14232
diff_mean_pitch1_2                   1.56      0.19     1.21     1.95 1.00    12505    11703
timbreStrings                       -0.03      0.09    -0.20     0.14 1.00    18748    13842
diff_mean_pitch1_2:timbreStrings    -0.10      0.18    -0.47     0.25 1.00    12472    12864
```

References

[1] Norman Carey and David Clampitt. Aspects of well-formed scales. *Music Theory Spectrum*, 11 (2):187–206, 1989.

[2] Adrienne L. Kaeppler, Amy Ku'uleialoha Stillman, Demeter Tsounis, Catherine Falk, Dorothy O'Donnell, Linda Barwick, Michael Ryan, Helen Reeves Lawrence, Don Niles, Rokucho Billy, Kim Bailey, Donald Brenneis, Ricardo D. Trimillos, Ted Salís, Lynn J. Martin, Judy Van Zile, Allan Thomas, and Jennie Coleman. *The Garland Encyclopedia of World Music: Australia and the Pacific Islands*, volume 9, chapter Introduction to Oceania and its Music: Musical Migrations, pages 54–69. Garland Publishing Inc., 1998.

[3] E. A. Smit, F. A. Dobrowohl, N. K. Schaal, Andrew J. Milne, and S. A. Herff. Perceived emotions of harmonic cadences. *Music & Science*, 3, 2020. doi: 10.1177/2059204320938635.

[4] H. S. Sarvasy. *A grammar of Nungon: A Papuan language of northeast New Guinea*. Brill, 2017.

[5] J. Wegmann and U. Wegmann. Yau anthropology background study. Unpublished manuscript, 1994.

[6] Adrienne L. Kaeppler and Don Niles. *The Garland Encyclopedia of World Music: Australia and the Pacific Islands*, volume 9, chapter The Music and Dance of New Guinea, pages 498–513. Garland Publishing Inc., 1998.

[7] Denis Crowdy. *Guitar Style, Open Tunings, and Stringband Music in Papua New Guinea*. Apwitihire: studies in Papua New Guinea musics. Institute of Papua New Guinea Studies, Boroko, Papua New Guinea, 2005.

[8] Michael Webb. *Palang* conformity and *fulset* freedom: Encountering Pentecostalism's "sensational" liturgical forms in the postmissionary church in Lae, Papua New Guinea. *Ethnomusicology*, 55(3):445–472, 2011.

[9] A. Capell. A typology of concept domination. *Lingua*, 15:451–462, 1950.

[10] B. S. Atal. Automatic speaker recognition based on pitch contours. *The Journal of the Acoustical Society of America*, 52(6B):1687–1697, 1972.

[11] Julius O. Smith III. Spectral audio signal processing: Quadratic interpolation of spectral peaks.

[12] Andrew J. Milne, William A. Sethares, Robin Laney, and David B. Sharp. Modelling the similarity of pitch collections with expectation tensors. *Journal of Mathematics and Music*, 5(1):1–20, may 2011.

[13] Nori Jacoby, Elizabeth Hellmuth Margulis, Martin Clayton, Erin Hannon, Henkjan Honing, John Iversen, Tobias Robert Klein, Samuel A. Mehr, Lara Pearson, Isabelle Peretz, Marc Perlman, Rainer Polak, Andrea Ravignani, Patrick E. Savage, Gavin Steingo, Catherine J. Stevens, Laurel Trainor, Sandra Trehub, Michael Veal, and Melanie Wald-Fuhrmann. Cross-cultural work in music cognition: Challenges, insights, and recommendations. *Music Perception*, 37(3):185–195, 2020. doi: 10.1525/mp.2020.37.3.185.

[14] G. Athanasopoulos, T. Eerola, I. Lahdelma, and M. Kaliakatsos-Papakostas. Harmonic organisation conveys both universal and culture-specific cues for emotional expression in music. *PLOS One*, 16(1):1–17, 2021. doi: 10.1371/journal.pone.0244964.
